# Leading mediators of sex differences in the incidence of dementia in community-dwelling adults in the UK Biobank: a retrospective cohort study

**DOI:** 10.1186/s13195-022-01140-2

**Published:** 2023-01-09

**Authors:** Xianwen Shang, Eddy Roccati, Zhuoting Zhu, Katerina Kiburg, Wei Wang, Yu Huang, Xueli Zhang, Xiayin Zhang, Jiahao Liu, Shulin Tang, Yijun Hu, Zongyuan Ge, Honghua Yu, Mingguang He

**Affiliations:** 1 Guangdong Eye Institute, Department of Ophthalmology, Guangdong Provincial People’s Hospital (Guangdong Academy of Medical Sciences), Southern Medical University, Guangzhou, 510080 China; 2 Guangdong Cardiovascular Institute, Guangdong Provincial People’s Hospital, Guangdong Academy of Medical Sciences, Guangzhou, 510080 China; 3 Centre for Eye Research Australia, Melbourne, VIC 3002 Australia; 4 Department of Medicine (Royal Melbourne Hospital), University of Melbourne, Melbourne, VIC 3050 Australia; 5 Wicking Dementia Research and Education Centre, University of Tasmania, Hobart, TAS 7001 Australia; 6 State Key Laboratory of Ophthalmology, Zhongshan Ophthalmic Center, Sun Yat-sen University, Guangzhou, 510060 China; 7 Monash e-Research Center, Faculty of Engineering, Airdoc Research, Nvidia AI Technology Research Center, Monash University, Melbourne, VIC 3800 Australia

**Keywords:** Sex, Dementia, Lifestyle, Multimorbidity risk score, Biomarker, Mediation analysis

## Abstract

**Background:**

Little is known regarding whether sex assigned at birth modifies the association between several predictive factors for dementia and the risk of dementia itself.

**Methods:**

Our retrospective cohort study included 214,670 men and 214,670 women matched by age at baseline from the UK Biobank. Baseline data were collected between 2006 and 2010, and incident dementia was ascertained using hospital inpatient or death records until January 2021. Mediation analysis was tested for 133 individual factors.

**Results:**

Over 5,117,381 person-years of follow-up, 5928 cases of incident all-cause dementia (452 cases of young-onset dementia, 5476 cases of late-onset dementia) were documented. Hazard ratios (95% CI) for all-cause, young-onset, and late-onset dementias associated with the male sex (female as reference) were 1.23 (1.17–1.29), 1.42 (1.18–1.71), and 1.21 (1.15–1.28), respectively. Out of 133 individual factors, the strongest mediators for the association between sex and incident dementia were multimorbidity risk score (percentage explained (95% CI): 62.1% (45.2–76.6%)), apolipoprotein A in the blood (25.5% (15.2–39.4%)), creatinine in urine (24.9% (16.1–36.5%)), low-density lipoprotein cholesterol in the blood (23.2% (16.2–32.1%)), and blood lymphocyte percentage (21.1% (14.5–29.5%)). Health-related conditions (percentage (95% CI) explained: 74.4% (51.3–88.9%)) and biomarkers (83.0% (37.5–97.5%)), but not lifestyle factors combined (30.1% (20.7–41.6%)), fully mediated sex differences in incident dementia. Health-related conditions combined were a stronger mediator for late-onset (75.4% (48.6–90.8%)) than for young-onset dementia (52.3% (25.8–77.6%)), whilst lifestyle factors combined were a stronger mediator for young-onset (42.3% (19.4–69.0%)) than for late-onset dementia (26.7% (17.1–39.2%)).

**Conclusions:**

Our analysis matched by age has demonstrated that men had a higher risk of all-cause, young-onset, and late-onset dementias than women. This association was fully mediated by health-related conditions or blood/urinary biomarkers and largely mediated by lifestyle factors. Our findings are important for understanding potential mechanisms of sex in dementia risk.

**Supplementary Information:**

The online version contains supplementary material available at 10.1186/s13195-022-01140-2.

## Introduction

Dementia is a neurocognitive disorder that is associated with significant functional deficits in language, behaviour, and cognition [1]. Cognitive impairment is associated with decreased functionality in activities of daily living among those with dementia [2]. The epidemic of dementia and its related disability-adjusted life year, mortality, and economic cost imposes a tremendous burden on the health care system globally [3–5]. Women are more likely to experience dementia than men [6], particularly at older ages [7, 8]. However, the underlying mechanisms for sex differences in dementia risk are largely unknown.

Many modifiable risk factors including lifestyle, chronic conditions, biomarkers, and air pollutants as well as non-modifiable risk factors such as apolipoprotein E epsilon 4 (APOE4) have been linked to dementia risk [9, 10]. Previous studies have investigated whether these risk factors modify the association between sex and dementia [6, 11, 12]. Recent evidence suggests sex hormones such as follicle-stimulating hormone, endogenous and exogenous oestrogen, luteinizing hormone, and testosterone may influence female susceptibility to dementia onset [12–14]. The interplay between genetic risk factors and sex as well as sex-specific changes at molecular levels in the brain, blood, or cerebrospinal fluid may also explain sex differences in dementia risk [15]. It is also possible that medical history and socioeconomic factors mediate the association between sex and dementia risk [16, 17]. However, how much sex differences in dementia risk could be explained by a wide range of individual determinants in isolation and in combination remains to be explored.

Identifying important mediators for sex differences in the incidence of dementia would facilitate understanding of underlying mechanisms. Using retrospective data from the UK Biobank cohort study, we sought to examine the association between sex and incident all-cause, young-onset, and late-onset dementias and whether a wide range of risk factors mediated this association.

## Methods

### Study population

The UK Biobank is a population-based cohort of more than 500,000 participants aged 40–73 years at baseline between 2006 and 2010 [18]. These participants attended one of the 22 assessment centres throughout the UK [18]. The study design and population have been detailed elsewhere [18]. Individuals with dementia or cognitive impairment at baseline were excluded from the analysis. To enable the comparability of lifestyle factors and biomarkers between genders, those of non-European ancestry were excluded. In addition, individuals without medical record linkage were excluded from the analysis. Notably, age is the most important determinant of dementia, and only adjusting for age might not be able to fully control the confounding due to age when comparing the difference in dementia risk by sex. Meanwhile, age is also highly related to lifestyle factors and biomarkers such that matching by age may help reduce the bias when exploring the mediation effects of these factors. Therefore, one woman for each man was matched by age at baseline (±1 year) in the analysis. Our study adhered to the AGReMA guidelines [19].

### Ascertainment of incident dementia

Dementia cases were ascertained using hospital inpatient records or death registers. Dementia was defined by a primary/secondary diagnosis using the international classification diseases coding system (detailed in Additional file 1: Table S1). Dementia was also defined as an underlying/contributory cause of death through linkage to death register data. Dementia diagnosed <65 years of age was categorized as young-onset dementia and that diagnosed ≥65 years was considered late-onset dementia [20]. The earliest recorded date was used as the onset date of dementia. Person-years were computed from the baseline assessment date to the date of onset dementia, date of death, or the end of follow-up (31 December 2020 for England and Wales and 31 January 2021 for Scotland), whichever came first.

### Sociodemographic data

Data on age, ethnicity, education, and household income were collected using a self-reported questionnaire on a touchscreen tablet. Townsend index of material deprivation was used to assess the neighbourhood-level socioeconomic status.

Sex (female/male) was self-reported. “Sex” refers to biological differences such as levels of hormones, whereas “gender” refers to differences in the impact of psychosocial and socioeconomic factors on biological markers between genders [21]. The effect of both “sex” and “gender” was involved in the present study, and “sex” was used in the text.

### Lifestyle factors

Self-reported data on lifestyle factors at baseline were collected via a touchscreen tablet. A short version of the International Physical Activity Questionnaire was used to estimate excess metabolic equivalent (MET)-hours/week of physical activity during work and leisure time. Intakes of food groups in the last year were self-reported using a structured questionnaire. A healthy diet score was calculated based on seven commonly eaten food groups (whole grains, refined grains, vegetable, fruit, fish, red meat, and processed meat) following recommendations on dietary priorities for cardiometabolic health [22]. A higher healthy diet score has been shown to be associated with a lower risk of dementia [23]. Sleep duration per day on average in the last 4 weeks was assessed with the survey item “About how many hours sleep do you get in every 24 h?” Alcohol consumption, as well as supplements including vitamins, folate, glucosamine, calcium, zinc, iron, and selenium per week in the last year, was self-reported.

### Genetic data

Affymetrix using a bespoke BiLEVE Axiom array or the UK Biobank Axiom array was used for genotyping [24]. All genetic data were quality controlled and imputed by the UK Biobank team. APOE genotype was directly genotyped based on two single-nucleotide polymorphisms (rs7412 and rs429358). APOE4+ dominant model of E3/E4 or E4/E4 was used to define the presence of APOE4.

### Blood tests

Blood samples were collected and analysed at a central laboratory at baseline between 2006 and 2010. Cholesterol was measured by direct enzymatic methods (Konelab, Thermo Fisher Scientific, Waltham, MA). Glycosylated haemoglobin (HbA1c) was measured using high-performance liquid chromatography. Serum cystatin C was measured by latex-enhanced immunoturbidimetric method on a Siemens ADVIA 1800 instrument. Serum 25-hydroxyvitamin D, a proxy for vitamin D levels, was measured using a chemiluminescent immunoassay (DiaSorin Liaison XL, DiaSorin Ltd., UK). Quality control was conducted by the UK Biobank central team (https://biobank.ctsu.ox.ac.uk/crystal/ukb/docs/serum_biochemistry.pdf).

### Urinary biomarker data

Urine assays for sodium, potassium, microalbumin, and creatinine were measured by ion-selective electrode analysis on a Beckman Coulter AU5400 (https://biobank.ndph.ox.ac.uk/showcase/ukb/docs/urine_assay.pdf).

### Health-related conditions

Chronic conditions at baseline were based on self-reported data or interviews. Participants were asked whether they had ever been told by a doctor that they had certain common medical conditions, such as cardiovascular disease, hypertension, diabetes, and depression. Additional disease cases at baseline were defined using inpatient data (initial diagnosis date before baseline interview date). Inpatient hospital data for the UK Biobank participants were available since 1997 [18]. Body mass index (BMI) was computed based on measured weight and height at baseline, and obesity was defined as BMI≥30 kg/m [2, 25]. A multimorbidity score was then computed based on 61 major diseases (Additional file 1: Table S2) [26].

### Familial medical history

The medical history of the father, mother, and siblings was collected using a touchscreen device. Medical conditions included heart disease, stroke, hypertension, diabetes, cancer, dementia, Parkinson’s disease, and depression.

### Environment measures

Air pollution and local environment measures were conducted by the Small Area Health Statistics Unit (http://www.sahsu.org/) and were linked centrally to the UK Biobank data (http://biobank.ctsu.ox.ac.uk/crystal/docs/EnviroExposEst.pdf). Particulate matter, nitrogen dioxide, and total nitrogen oxides were measured as annual average values in microgrammes per cubic metre. Road traffic measures were provided for the year 2008 from the Road Traffic Statistics Branch at the Department for Transport attached to the local road network; traffic data for unmonitored links were estimated based on surrounding monitored links. Data were also available regarding noise pollution, such as daytime, evening, and night-time average level of noise pollution (dB).

### Statistical analysis

Baseline characteristics were expressed as frequency (percentage) and means±standard deviations (SDs). *T*-test for continuous variables and chi-square for categorical variables were used to test the difference of between sexes. Cox regression models were conducted to examine the sex effect on the incidence of all-cause, young-onset, and late-onset dementias adjusted for age.

The potential mediation effects of a wide range of individual factors on the association between sex and incident dementia were estimated using Cox proportional hazards regression models adjusted for age [27]. We used the following criteria to establish mediation [27]: (1) the mediator was significantly associated with sex; (2) sex was significantly associated with dementia; (3) the mediator was significantly associated with dementia; and (4) the association between sex and dementia was attenuated by the mediator (Additional file 1: Fig. S1). Potential mediators examined included socioeconomic factors (*n*=3), lifestyle factors (*n*=19), health-related conditions (*n*=3), familial history of medical conditions (*n*=24), genetics (*n*=1), blood biomarkers (*n*=49), urinary biomarkers (*n*=4), and pollution measures (*n*=30, Additional file 1: Table S3). We also examined the mediation effect of these groups of factors combined. Whether individual chronic conditions used to create multimorbidity risk score mediated the association between sex and dementia was also tested. The mediation analysis was conducted using macro programmes created by Spiegelman et al. [28] Benjamin-Hochberg’s procedure was used to control the false discovery rate at a 5% level for multiple comparisons [29].

A sensitivity analysis was conducted to examine whether the important determinants mediated the association between sex and incident dementia by excluding those cases diagnosed in the first 5 years of follow-up. A further sensitivity analysis was conducted to test mediation associations among individuals with complete data.

Multiple imputations for missing data were conducted, and all covariates were included in the imputation models to create 5 imputed datasets.

Data analyses were conducted using SAS 9.4 for Windows (SAS Institute Inc.) and all *P* values were two-sided with statistical significance set at <0.05.

## Results

### Population selection and baseline characteristics

Baseline data were collected among 502,505 participants. After excluding individuals of non-European ancestry (*n*=30,380), those who could not be linked to inpatient data (*n*=27), those with prevalent dementia (*n*=345) or cognitive impairment (*n*=232), or those who developed dementia in the first year of follow-up (*n*=36), 471,485 participants were included in the matching analysis. After excluding those women who were not matched in pairs, 429,340 participants (214,670 men, 214,670 age-matched women) aged 40–71 years (mean ± SD: 57.0±8.1) were included in the final analysis (Additional file 1: Fig. S2). Women were more likely to have a lower income, to be less physically active, and to be non-smokers than men. Women had a higher healthy diet score and lower multimorbidity risk score, BMI, and HbA1c (Table 1). Women had higher apolipoprotein A, but lower creatinine in blood and urine than men (Additional file 1: Table S4). Percentages of participants with missing values in each variable and values in imputed and non-imputed data are listed in Additional file 1: Tables S5 and S6.

**Table 1:** Baseline characteristics in women and men

	Women	Men	*P*-value^a^
Age (years)	57.0 ± 8.1	57.0 ± 8.1	0.85
APOE4 carrier^b^			0.0027
No	16,3451 (76.1)	164,287 (76.5)	
Yes	51,219 (23.9)	50,383 (23.5)	
Education			0.39
College/university degree	66,142 (30.8)	72,277 (33.7)	
Upper secondary	25,972 (12.1)	22,704 (10.6)	
Final stage of secondary education	51,428 (24.0)	41,114 (19.2)	
Lower secondary	11,151 (5.2)	11,728 (5.5)	
First stage of secondary education	9202 (4.3)	19,614 (9.1)	
Vocational qualifications	12,300 (5.7)	9617 (4.5)	
None of above	38,475 (17.9)	37,616 (17.5)	
Household income (pounds)			<0.0001
<18,000	53,921 (25.1)	42,798 (19.9)	
18,000–30,999	63,354 (29.5)	55,153 (25.7)	
31,000–51,999	54,080 (25.2)	59,039 (27.5)	
52,000–100,000	34,999 (16.3)	45,899 (21.4)	
>100,000	8316 (3.9)	11,781 (5.5)	
Townsend index	−1.49 ± 2.94	−1.41 ± 3.05	<0.0001
Alcohol consumption			<0.0001
Never	9871 (4.6)	3782 (1.8)	
Previous	7821 (3.6)	7146 (3.3)	
Current	196,978 (91.8)	203,742 (94.9)	
Smoking			<0.0001
Never	125,641 (58.5)	103,859 (48.4)	
Former	70,067 (32.6)	84,483 (39.4)	
Current	18,962 (8.8)	26,328 (12.3)	
Physical activity (MET-minutes/week)	2561 ± 2179	2795 ± 2712	<0.0001
Diet score^c^	4.20 ± 1.38	3.49 ± 1.40	<0.0001
Sleep duration (hours)	7.19 ± 1.10	7.14 ± 1.08	<0.0001
BMI (kg/m^2^)	27.04 ± 5.08	27.84 ± 4.19	<0.0001
Overall health rating			<0.0001
Excellent	37,162 (17.3)	34,003 (15.8)	
Good	128,906 (60.0)	120,844 (56.3)	
Fair	40,750 (19.0)	48,891 (22.8)	
Poor	7852 (3.7)	10,932 (5.1)	
Long-standing illness, disability, or infirmity			<0.0001
No	149,552 (69.7)	137,645 (64.1)	
Yes	65,118 (30.3)	77,025 (35.9)	
Multimorbidity risk score	0.24 ± 0.27	0.30 ± 0.32	<0.0001
Trunk fat percentage	34.13 ± 7.77	27.62 ± 6.71	<0.0001
Whole body fat percentage	36.58 ± 6.87	25.28 ± 5.82	<0.0001
HbA1c (mmol/mol)	13.52 ± 0.93	15.00 ± 1.00	<0.0001
Triglycerides (mmol/L)	1.58 ± 0.83	1.97 ± 1.12	<0.0001
HDL-C (mmol/L)	1.58 ± 0.35	1.30 ± 0.30	<0.0001
LDL-C (mmol/L)	3.63 ± 0.84	3.49 ± 0.83	<0.0001

Data are mean (standard deviation) or *N* (%). *APOE4*, apolipoprotein E4; *BMI*, body mass index; *HbA1c*, glycated haemoglobin; *HDL-C*, high-density lipoprotein cholesterol; *LDL-C*, low-density lipoprotein cholesterol; *MET*, metabolic equivalent

^a^*T*-test was used to test the difference of continuous variables between sexes and chi-square for categorical variables

^b^APOE4+ dominant model of E3/E4 and E4/E4 was used to define the presence of APOE4

^c^Diet score was computed based on seven commonly eaten food groups following recommendations on dietary priorities for cardiometabolic health with a higher score representing a healthier diet

### Incidence of dementia

**Fig. 1:**
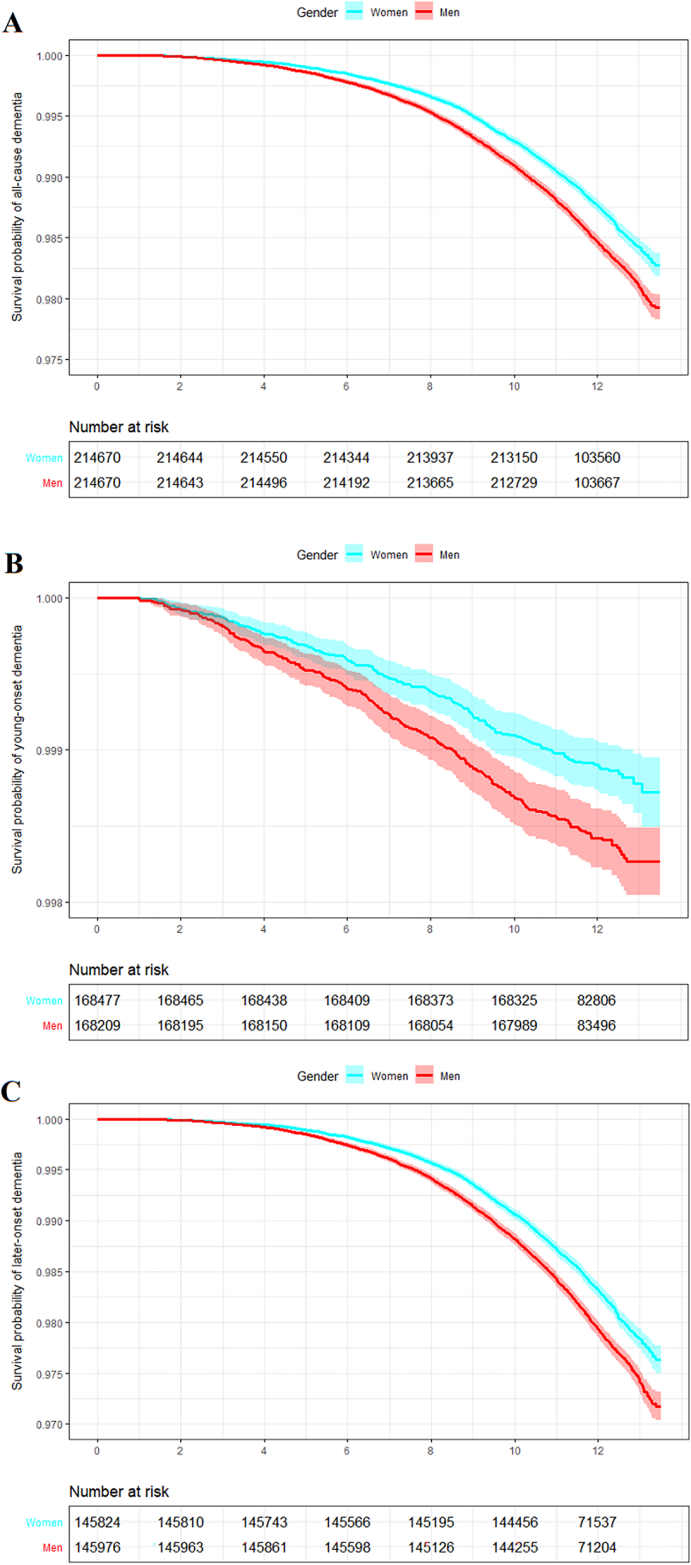
Survival probability of dementia in women and men. Panels **A**, **B**, and **C** display the survival probability of all-cause, young-onset, and late-onset dementias in women and men

Over 5,117,381 person-years of follow-up, 5928 cases of incident all-cause dementia were documented. Over 4,029,006 person-years of follow-up, 452 cases of young-onset dementia were documented. Over 3,478,484 person-years of follow-up, 5476 cases of late-onset dementia were documented. For all-cause, young-onset, and late-onset dementias, women had a higher survival probability than men. The aged-adjusted hazard ratios ([HRs] 95% CIs) for all-cause, young-onset, and late-onset dementias associated with the male sex were 1.23 (1.17–1.29), 1.42 (1.18–1.71), and 1.21 (1.15–1.28), respectively (Fig. 1). Men had a higher risk of Alzheimer’s disease (HR (95% CI): 1.14 (1.05–1.24)) and vascular dementia (1.48 (1.32–1.66)) compared with women after adjustment for age.

### Mediators of sex differences in all-cause dementia

**Fig. 2:**
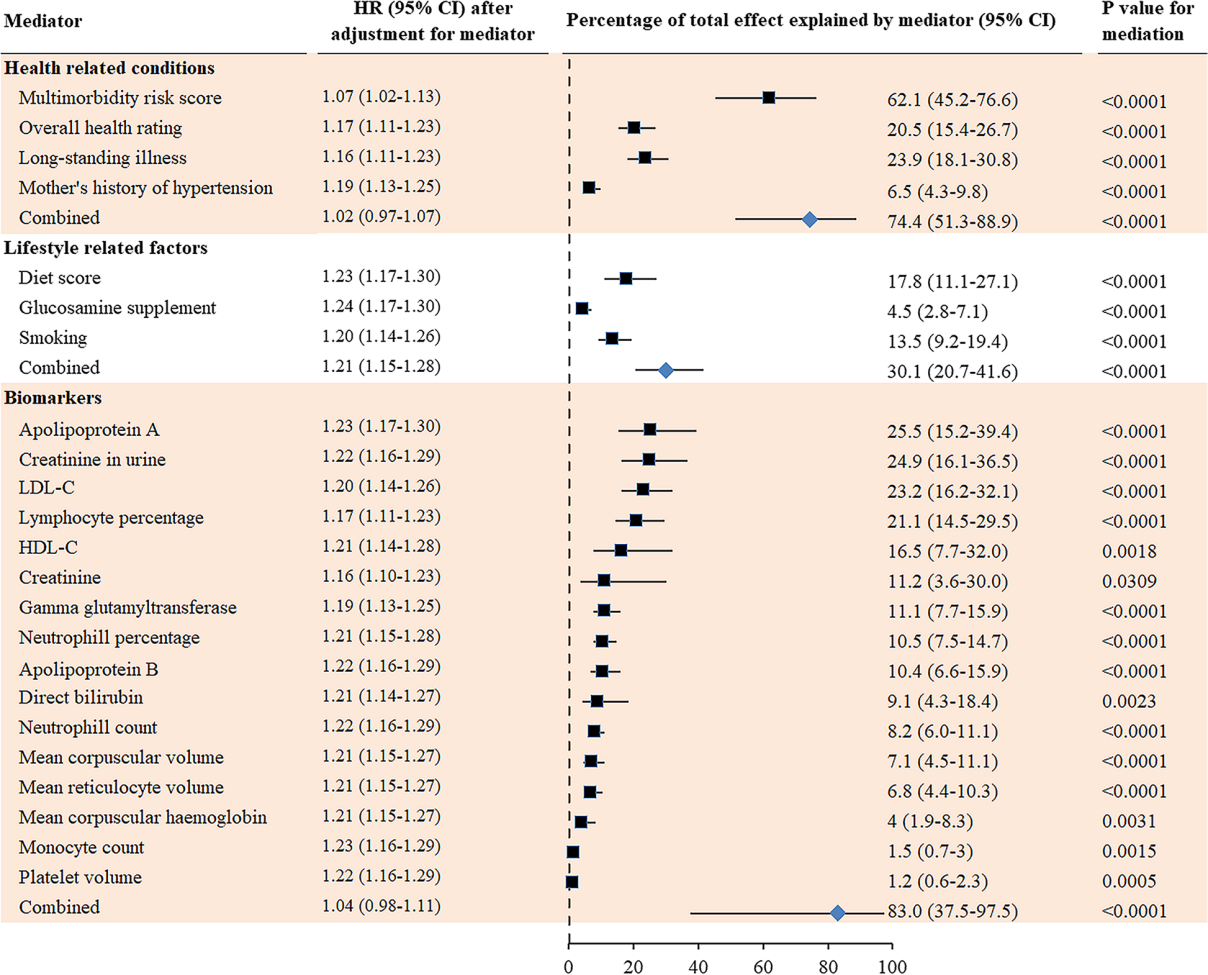
Mediators for sex differences in the incidence of all-cause dementia. Cox proportional hazards regression models were used to estimate the potential mediation effects of 133 individual factors on the association between sex and incidence of all-cause dementia. HR adjusted for mediator shows whether the association between sex and dementia was attenuated by the mediator (criterion 4). Results for the test of the other three criteria are shown in Tables 1 and S4 (criterion 1), Fig. 1 (criterion 2), and Table S7 (criterion 3)

The strongest mediators for the association between sex and incident dementia were multimorbidity risk score (percentage explained (95% CI): 62.1% (45.2–76.6%)), apolipoprotein A in the blood (25.5% (15.2–39.4%)), creatinine in urine (24.9% (16.1–36.5%)), LDL-C in the blood (23.2% (16.2–32.1%)), and blood lymphocyte percentage (21.1% (14.5–29.5%)). Health-related conditions (74.4% (51.3–88.9%)), or biomarkers (83.0% (37.5–97.5%)), but not lifestyle factors combined (30.1% (41.6–20.7%)) fully mediated sex differences in incident dementia (Fig. 2). For individual chronic diseases used to create multimorbidity risk score, coronary heart disease (27.4% (20.0–36.2%)), diabetes (19.2% (14.2–25.5%)), hearing impairment (17.8% (12.7–24.5%)), high cholesterol (14.2% (10.3–19.2%)), and hypertension (11.2% (8.2–15.2%)) were the strongest mediators (Additional file 1: Fig. S3). The results on the association between induvial mediators and incident dementia are shown in Additional file 1: Table S7.

### Mediators of sex differences in young-onset dementia

**Fig. 3:**
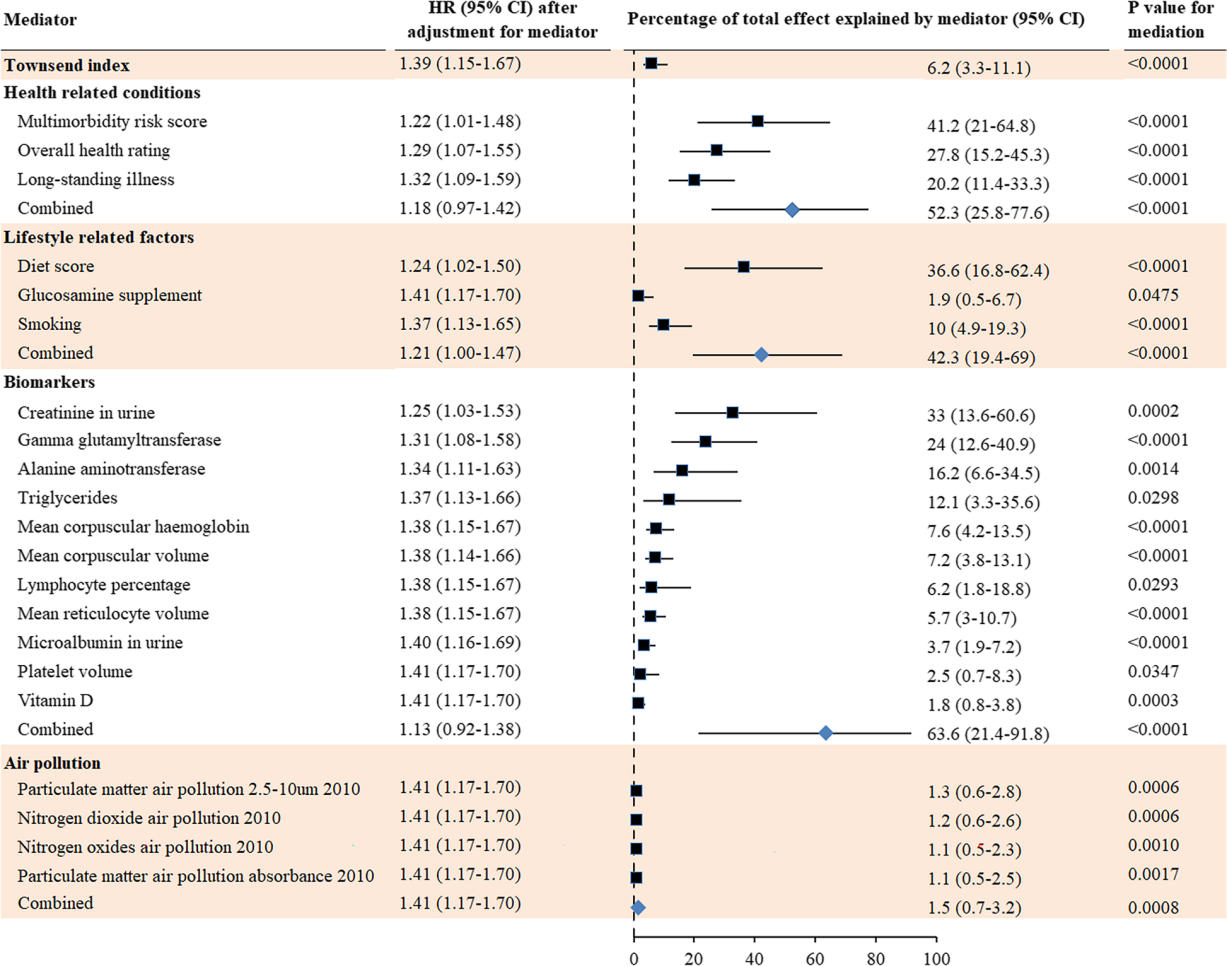
Mediators for sex differences in the incidence of young-onset dementia. Dementia diagnosed <65 years of age was categorized as young-onset dementia. Cox proportional hazards regression models were used to estimate the potential mediation effects of 133 individual factors on the association between sex and incidence of young-onset dementia. HR adjusted for mediator shows whether the association between sex and dementia was attenuated by the mediator (criterion 4). Results for the test of the other three criteria are shown in Tables 1 and S4 (criterion 1), Fig. 1 (criterion 2), and Table S7 (criterion 3)

Percentages (95% CIs) of sex differences in the incidence of young-onset dementia explained by multimorbidity risk score, diet score, creatinine in urine, overall health rating, and gamma glutamyltransferase in blood were 41.2% (21–64.8%), 36.6% (16.8–62.4%), 33.0% (13.6–60.6%), 27.8% (15.2–45.3%), and 24.0% (12.6–40.9%), respectively. Health-related conditions (52.3% (25.8–77.6%)), lifestyle factors (42.3% (19.4–69.0%)), or biomarkers combined (63.6% (21.4–91.8%)) fully mediated the association between sex and incident young-onset dementia. Townsend index and air pollution were also significant mediators (Fig. 3).

Coronary heart disease (17.7% (8.4–33.6%)), hearing impairment (16.6% (8.6–29.6%)), alcohol problems (13.8% (6.6–26.5%)), hypertension (12.5% (6.1–23.8%)), and high cholesterol (12.2% (5.4–25%)) were the leading mediators (Additional file 1: Fig. S4).

### Mediators of sex differences in late-onset dementia

**Fig. 4:**
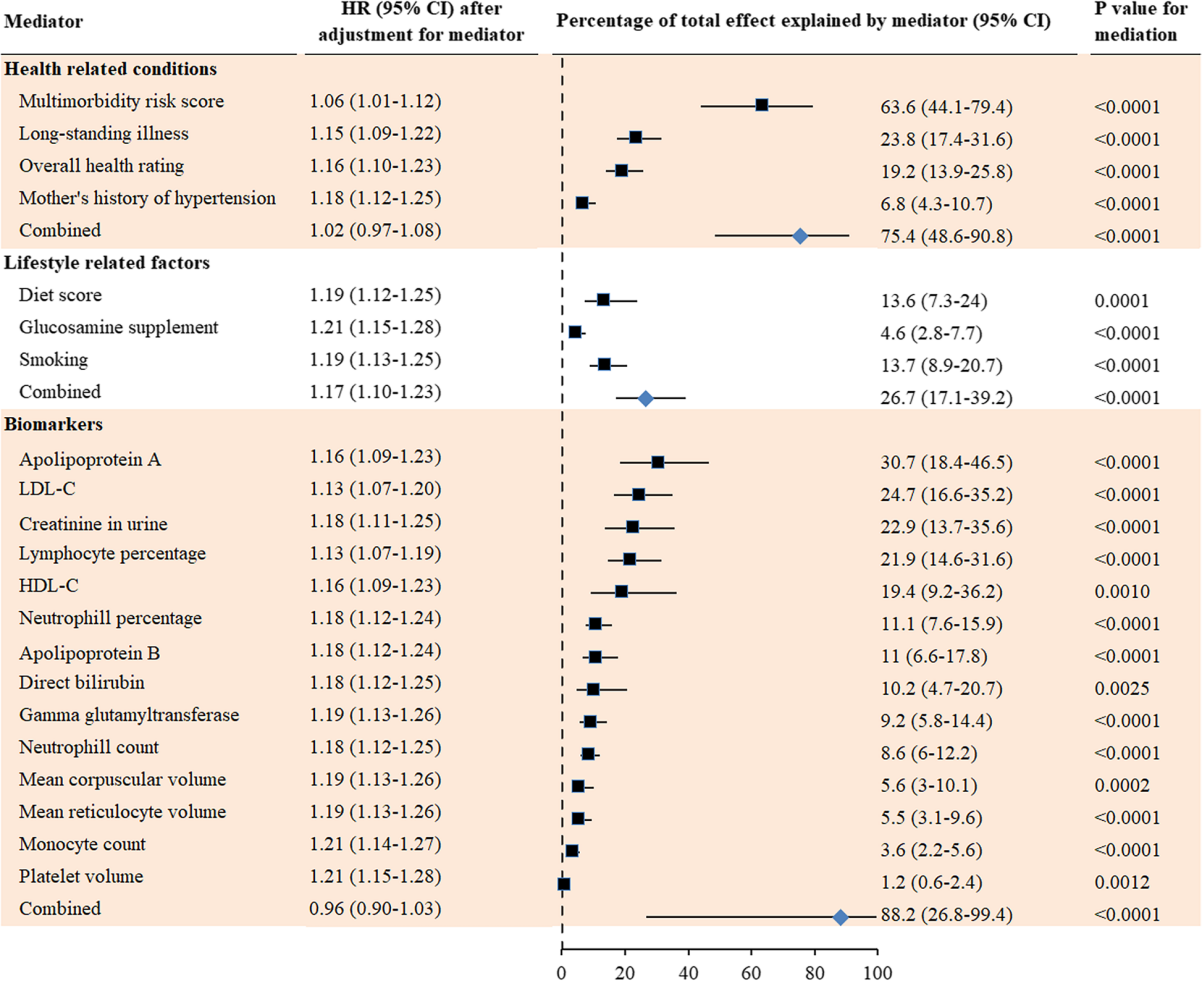
Mediators for sex differences in the incidence of late-onset dementia. Dementia diagnosed ≥65 years of age was considered late-onset dementia. Cox proportional hazards regression models were used to estimate the potential mediation effects of 133 individual factors on the association between sex and incidence of late-onset dementia. HR adjusted for mediator shows whether the association between sex and dementia was attenuated by the mediator (criterion 4). Results for the test of the other three criteria are shown in Tables 1 and S4 (criterion 1), Fig. 1 (criterion 2), and Table S7 (criterion 3)

Multimorbidity risk score was the leading mediator for sex differences in the incidence of late-onset dementia (63.6% (44.1–79.4%)). Health-related conditions combined fully mediated the association between sex and late-onset dementia. The percentage (95% CI) of sex differences in late-onset dementia mediated by lifestyle factors combined was 26.7% (17.1–39.2%). Biomarkers combined fully mediated the association between sex and incident late-onset dementia (88.2% (26.8–99.4%), Fig. 4).

Of the individual chronic diseases investigated, coronary heart disease (28.5% (20.1–38.2%)), diabetes (20.4% (14.6–27.7%)), hearing impairment (17.6% (11.9–25.2%)), high cholesterol (14.5% (10.2–20.3%)), and hypertension (11.2% (7.9–15.6%)) largely mediated the association between sex and risk of late-onset dementia (Additional file 1: Fig. S5).

### Sensitivity analysis

Mediation analysis among individuals by excluding dementia cases diagnosed in the first five of follow-up showed that health-related conditions (74.6% (48.5–90.2%)), lifestyle factors (64.8% (44.7–80.7%)), or biomarkers combined (93.8% (5.2–100%)) fully mediated sex differences in incident dementia (Additional file 1: Fig. S6). Similar results were found among those with complete data compared to those of the main findings (Additional file 1: Fig. S7).

## Discussion

In this large population of community-dwelling adults, men had a higher risk of all-cause dementia than women and this association was stronger for young-onset dementia than for late-onset dementia. Multimorbidity risk score, apolipoprotein A, creatinine in urine, LDL-C, and lymphocyte percentage were the leading mediators for sex differences in incident dementia. Health-related conditions and biomarkers combined fully mediated sex differences in incident dementia. Lifestyle factors explained more sex differences in young-onset than in late-onset dementia.

Around two-thirds of adults living with dementia are women [30], and this is possibly due to the fact that women live longer, on average, than men [31]. However, our analysis matched by age has demonstrated that men had a higher risk of dementia than women independent of age. This indicates sex differences in the incidence of dementia may be due to other mechanisms rather than ageing. It has been reported that the risk for Alzheimer’s disease associated with APOE Ɛ4/Ɛ4 was higher in men than in women [32]. Men had a higher risk of dementia than women possibly due to the observed worse memory in men than in women overall and more specifically beyond age 40 years [33]. A recent cohort study has demonstrated that education might be more likely to act as a contributor to cognitive reserve in women than in men [34]. These findings may partly explain why men than women were more prone to develop dementia in later life. Compared with women, men had a higher degree of cerebral amyloid angiopathy [35], higher uptake of Pittsburgh compound B [36], and greater age-related brain atrophy in frontal, parietal, and temporal regions [33], which may also partly explain the higher incidence of dementia in men than in women. A recent meta-analysis showed that women had a higher prevalence of moderate to severe neuropsychiatric symptoms whereas men had a higher prevalence of more severe apathy [37]. Another recent meta-analysis has shown that the prevalence of young-onset dementia was 293.1 per 100,000 population for men and 216.5 per 100,000 population for women [20]. We found the risk associated with the female sex was larger for young-onset dementia than for late-onset dementia. This needs to be confirmed by future cohort studies.

The importance of lifestyle factors including diet, physical activity, smoking, and alcohol consumption on the development of dementia has been highlighted in previous studies [9, 11, 12]. Recent research has investigated whether the association between lifestyle factors and dementia differed between sexes [11, 12]. We found lifestyle factors largely mediated the association between sex and incident dementia and even fully mediated the association between sex and incident young-onset dementia. It is possible that women had healthier lifestyle habits such as never smoking and higher diet quality such that they were less likely to develop dementia especially young-onset dementia. Although whether sex differences in the incidence of dementia are mediated by lifestyle factors has not been reported in previous studies, our findings are supported by research reporting that a healthy diet is particularly protective of dementia for women [38].

Strong evidence has demonstrated the importance of chronic diseases and their multimorbidity in the development of dementia [9, 26]. A previous study has investigated the associations between cardiovascular risk factors and dementia stratified by sex [39]; however, whether the association between sex and incident dementia was mediated by chronic diseases remains to be explored. We found that a multimorbidity risk score was the most important individual mediator for the association between sex and incident dementia. In our study, men had higher multimorbidity risk score than women and multimorbidity risk score was an important risk factor for dementia. This explained why multimorbidity risk score largely mediated the association between sex and dementia risk. For individual chronic conditions used to create multimorbidity risk score, coronary heart disease was the leading mediator followed by other cardiometabolic disorders including diabetes, high cholesterol, hypertension, stroke, heart failure, and atrial fibrillation in our study. Hearing impairment, alcohol problems, and Parkinson’s disease also play an important role in sex differences in incident dementia. Although the effect size is small, mother’s history of hypertension was a significant mediator for the association between sex and incident dementia. However, it is unknown whether individuals were born before or after the development of hypertension among their mothers. Therefore, mother’s history of hypertension may not be a reliable mediator. Our research highlights the importance of the prevention or treatment of specific diseases to fill the gap in dementia risk between sexes.

The role of biomarkers in the development of dementia should not be overlooked. We found lipid-related factors including apolipoprotein A, apolipoprotein B, LDL-C, and HDL-C, which are highly correlated with cardiometabolic disorders, were significant mediators of the association between sex and incident dementia. This was consistent with our results for cardiometabolic disorders as leading important mediators as sex differences in incident dementia. The mediation effect of these biomarkers on sex difference in dementia risk may be partly attributable to the fact that the male sex was associated with adverse levels of biomarkers and these biomarkers were strongly associated with dementia risk as shown in our analysis. Similarly, recent studies have shown that the potential effect of lipids in the development of dementia may differ between sexes [40, 41]. We found both blood and urinary creatinine mediated the association between sex and dementia. This is in line with a cohort study showing that the association between glomerular filtration rate and dementia risk was significant in women only [42]. Some other biomarkers also slightly mediated sex differences in incident dementia, but this needs to be supported by follow-up studies. Although individual biomarkers did not contribute much to the association between sex and dementia, these biomarkers combined fully mediated the association. This suggests it is critical to monitor, manage, and control important biomarkers for the prevention of dementia.

To our knowledge, this is the first study to investigate important mediators from a wide range of factors for the association between sex and incident dementia. The present study has several limitations. Firstly, some cases of all-cause dementia may not be captured in the medical records or death registers [43]. However, more recent studies have shown that routine health care data can achieve a high accuracy of dementia ascertainment [43, 44]. Notably, previous research has shown that positive predictive value for all-cause dementia was 87.3% and 80.0% for hospital admissions and mortality data respectively in the UK Biobank [45]. Secondly, dementia might have occurred before the diagnosis using inpatient data, as the prodromal period of dementia can last many years [46]. Therefore, dementia might have occurred before the assessment of mediators. However, our sensitivity analysis by excluding dementia cases developed in the first 5 years of follow-up showed similar results to the main findings, which may have reduced the possibility that mediators occurred before the diagnosis of dementia. Thirdly, although sex-related hormones including sex hormone-binding globulin, testosterone, and oestradiol were analysed as mediators, data on potential changes in sex assigned at birth and gender identity were not available in our study. Fourthly, pregnancy complications including preeclampsia have been linked to dementia risk [47], but pregnancy complications and reproductive health were not included in our analysis given the unavailability of data. Fifthly, some unmatched individuals were excluded from the analysis, which might have resulted in lower precision of estimates compared to using the whole cohort. However, a low exclusion rate (8.9%) would not impact the representativeness of the cohort. Finally, our analysis was restricted to a subgroup of the UK Biobank cohort of European ancestry. Thus, our findings may not be generalized to other ethnic groups.

In conclusion, men have a higher risk of all-cause, young-onset, and late-onset dementias than women. This association is fully mediated by health-related conditions and blood/urinary biomarkers and largely mediated by lifestyle factors. Lifestyle factors are stronger mediators for young-onset dementia whilst health-related conditions are stronger mediators for late-onset dementia. The association between sex and young-onset dementia is also slightly mediated by the Townsend index or air pollution.

## Supplementary Information

**Additional file 1: Figure S1.** Diagram for pathways in the mediation analysis. **Figure S2.** Flowchart for population selection from the UK Biobank. **Figure S3.** Sex differences in the incidence of all-cause dementia mediated by individual chronic diseases. **Figure S4.** Sex differences in the incidence of young-onset dementia mediated by individual chronic diseases. **Figure S5.** Sex differences in the incidence of late-onset dementia mediated by individual chronic diseases. **Figure S6.** Mediators of sex differences in the incidence of dementia among individuals by excluding those who were diagnosed with dementia in the first five years of follow-up. **Figure S7.** Mediators of sex differences in the incidence of dementia among individuals with complete data. **Table S1.** Codes for international classification disease and self-reported fields for dementia. **Table S2.** Chronic conditions used to create the multimorbidity score for dementia. **Table S3.** Potential mediators tested in the analysis. **Table S4.** Other baseline characteristics in women and men. **Table S5.** Categorical variables in imputed and non-imputed data. **Table S6.** Continuous variables in imputed and non-imputed data. **Table S7.** Risk for incident dementia associated with mediators.

## Data Availability

Data are available in a public, open-access repository (https://www.ukbiobank.ac.uk/).
