# Supplementary material for: Leading mediators of sex differences in the incidence of dementia in community-dwelling adults in the UK Biobank: a retrospective cohort study
Source: Alzheimers Res Ther. 2023 Jan 9;15:7. doi: 10.1186/s13195-022-01140-2 (PMC9827665; doi:10.1186/s13195-022-01140-2)
Supplement: Supplementary file 1 — Additional file 1: Figure S1. Diagram for pathways in the mediation analysis. Figure S2. Flowchart for population selection from the UK Biobank. Figure S3. Sex differences in the incidence of all-cause dementia mediated by individual chronic diseases. Figure S4. Sex differences in the incidence of young-onset dementia mediated by individual chronic diseases. Figure S5. Sex differences in the incidence of late-onset dementia mediated by individual chronic diseases. Figure S6. Mediators of sex differences in the incidence of dementia among individuals by excluding those who were diagnosed with dementia in the first five years of follow-up. Figure S7. Mediators of sex differences in the incidence of dementia among individuals with complete data. Table S1. Codes for international classification disease and self-reported fields for dementia. Table S2. Chronic conditions used to create the multimorbidity score for dementia. Table S3. Potential mediators tested in the analysis. Table S4. Other baseline characteristics in women and men. Table S5. Categorical variables in imputed and non-imputed data. Table S6. Continuous variables in imputed and non-imputed data. Table S7. Risk for incident dementia associated with mediators. [file 13195_2022_1140_MOESM1_ESM.docx]

**Additional file 1**

**Figure S1. Diagram for pathways in the mediation analysis**

**Figure S2. Flowchart for population selection from the UK Biobank**

**Figure S3. Sex differences in the incidence of all-cause dementia mediated by individual chronic diseases**

**Figure S4. Sex differences in the incidence of young-onset dementia mediated by individual chronic diseases**

**Figure S5. Sex differences in the incidence of late-onset dementia mediated by individual chronic diseases**

**Figure S6. Mediators of sex differences in the incidence of dementia among individuals by excluding those who were diagnosed with dementia in the first five years of follow-up**

**Figure S7. Mediators of sex differences in the incidence of dementia among individuals with complete data**

**Table S1. Codes for international classification disease and self-reported fields for dementia**

**Table S2. Chronic conditions used to create the multimorbidity score for dementia**

**Table S3. Potential mediators tested in the analysis**

**Table S4. Other baseline characteristics in women and men**

**Table S5. Categorical variables in imputed and non-imputed data**

**Table S6. Continuous variables in imputed and non-imputed data**

**Table S7. Risk for incident dementia associated with mediators**


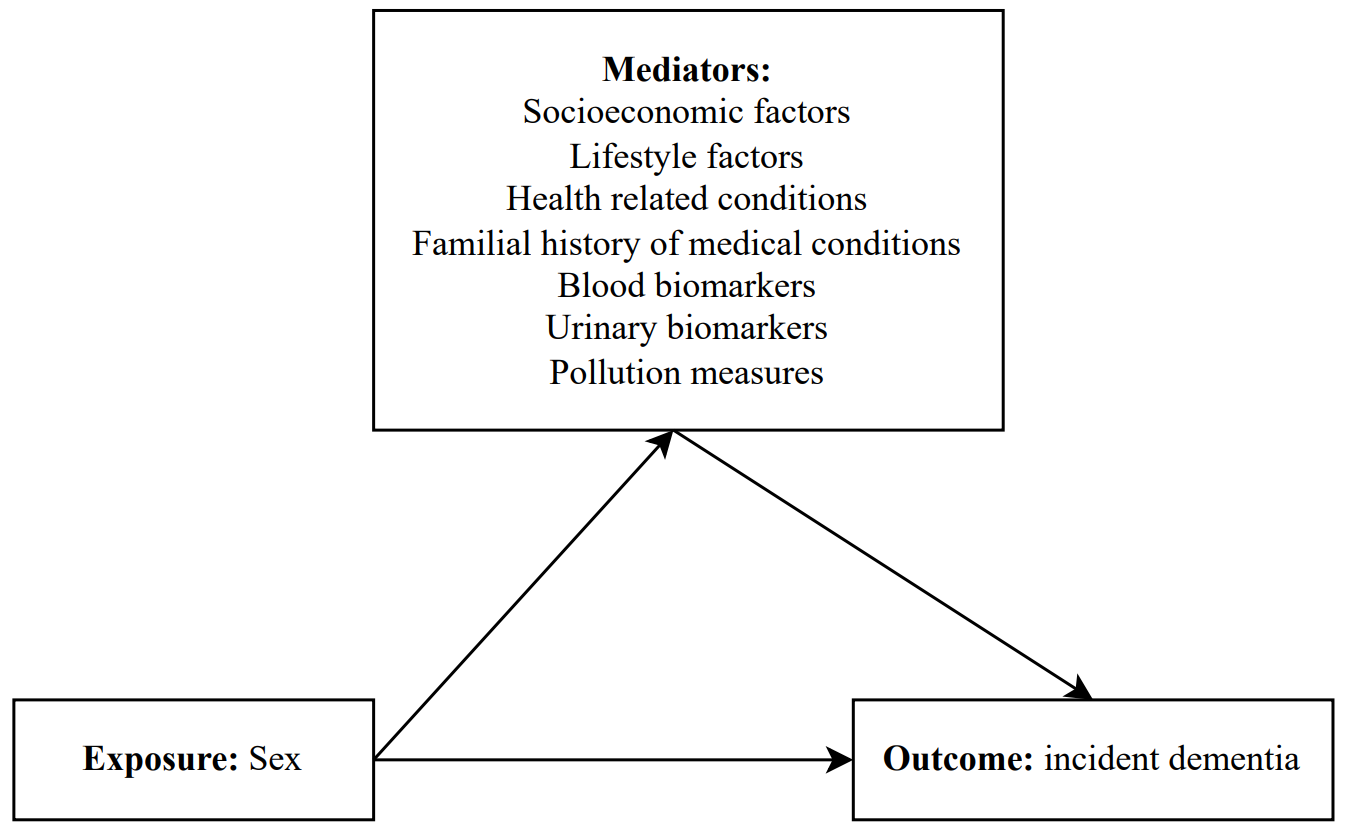


**Figure S1. Diagram for pathways in the mediation analysis**


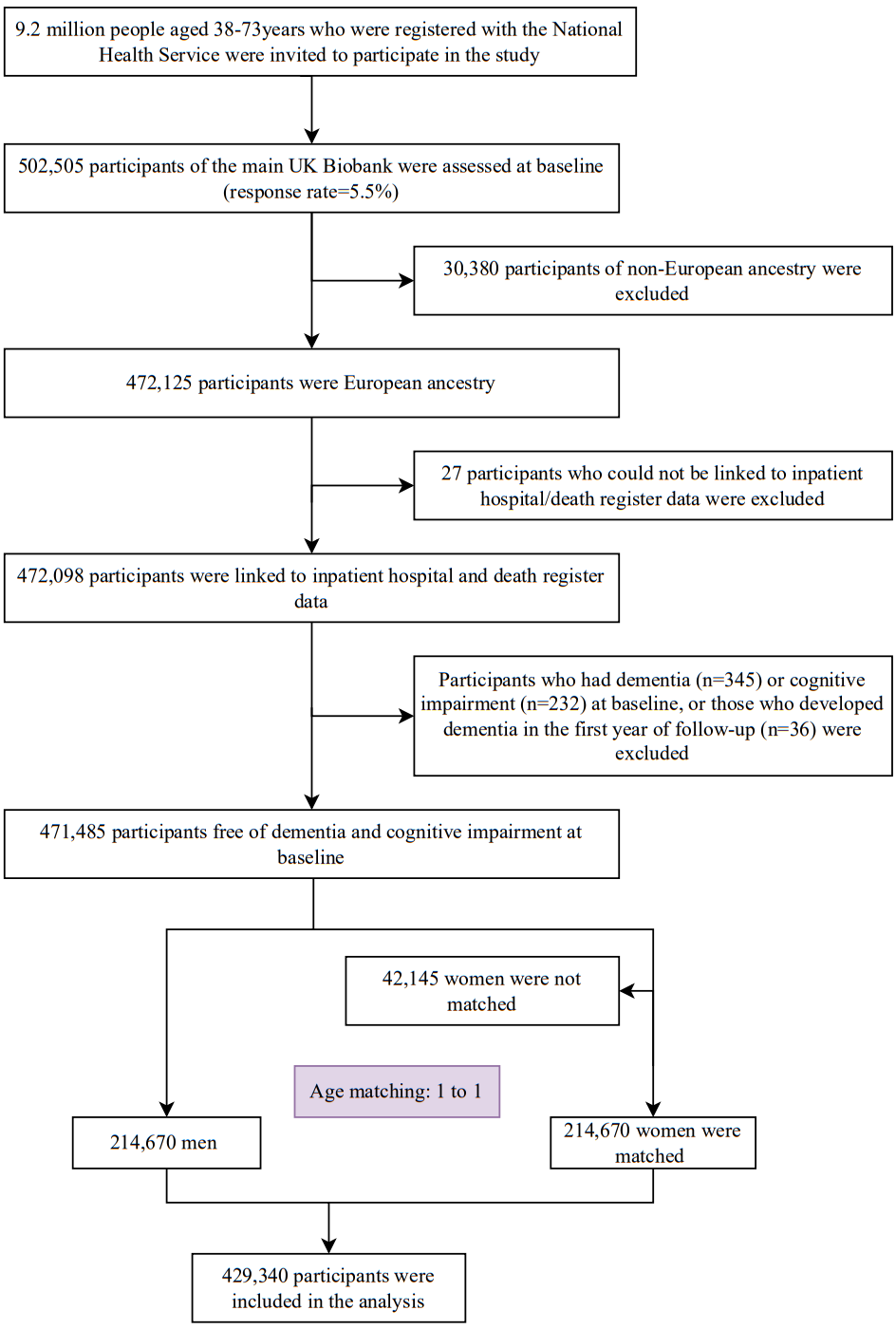


**Figure S2. Flowchart for population selection from the UK Biobank**

**Figure S3. Sex differences in the incidence of all-cause dementia mediated by individual chronic diseases**

CI, confidence interval; COPD, chronic obstructive pulmonary disease; HR, hazard ratio.

Cox proportional hazards regression models were used to estimate the potential mediation effects of 63 individual chronic conditions (used to create multimorbidity risk score) on the association between sex and incidence of all-cause dementia. Mediation was established using the following criteria: (1) the mediator was significantly associated with sex; (2) sex was significantly associated with dementia; (3) the mediator was significantly associated with dementia; and (4) the association between sex and dementia was attenuated by the mediator. Benjamin-Hochberg's procedure was used to control the false discovery rate at a 5% level for multiple comparisons. The figure only shows the results with significant mediation effects.

**Figure S4. Sex differences in the incidence of young-onset dementia mediated by individual chronic diseases**

CI, confidence interval; COPD, chronic obstructive pulmonary disease; HR, hazard ratio.

Cox proportional hazards regression models were used to estimate the potential mediation effects of 63 individual chronic conditions (used to create multimorbidity risk score) on the association between sex and incidence of young-onset dementia. Mediation was established using the following criteria: (1) the mediator was significantly associated with sex; (2) sex was significantly associated with dementia; (3) the mediator was significantly associated with dementia; and (4) the association between sex and dementia was attenuated by the mediator. Benjamin-Hochberg's procedure was used to control the false discovery rate at a 5% level for multiple comparisons. The figure only shows the results with significant mediation effects.

**Figure S5. Sex differences in the incidence of late-onset dementia mediated by individual chronic diseases**

CI, confidence interval; COPD, chronic obstructive pulmonary disease; HR, hazard ratio.

Cox proportional hazards regression models were used to estimate the potential mediation effects of 63 individual chronic conditions (used to create multimorbidity risk score) on the association between sex and incidence of late-onset dementia. Mediation was established using the following criteria: (1) the mediator was significantly associated with sex; (2) sex was significantly associated with dementia; (3) the mediator was significantly associated with dementia; and (4) the association between sex and dementia was attenuated by the mediator. Benjamin-Hochberg's procedure was used to control the false discovery rate at a 5% level for multiple comparisons. The figure only shows the results with significant mediation effects.

**Figure S6.** **Mediators of sex differences in the incidence of dementia among individuals by excluding those who were diagnosed with dementia in the first five years of follow-up**

CI, confidence interval; HR, hazard ratio; HDL-C, high-density lipoprotein cholesterol; LDL-C, low-density lipoprotein cholesterol.

A sensitivity analysis was conducted among individuals by excluding those who were diagnosed with dementia in the first five years of follow-up. Cox proportional hazards regression models were used to estimate the potential mediation effects of 128 individual factors on the association between sex and incidence of all-cause dementia. Mediation was established using the following criteria: (1) the mediator was significantly associated with sex; (2) sex was significantly associated with dementia; (3) the mediator was significantly associated with dementia; and (4) the association between sex and dementia was attenuated by the mediator. The mediation effects of main groups of factors combined were also tested. Benjamin-Hochberg's procedure was used to control the false discovery rate at a 5% level for multiple comparisons. The figure only shows the results with significant mediation effects.

**Figure S7. Mediators of sex differences in the incidence of dementia among individuals with complete data**

CI, confidence interval; HR, hazard ratio; HDL-C, high-density lipoprotein cholesterol; LDL-C, low-density lipoprotein cholesterol.

A sensitivity analysis was conducted among individuals with complete data. Cox proportional hazards regression models were used to estimate the potential mediation effects of 128 individual factors on the association between sex and incidence of all-cause dementia. Mediation was established using the following criteria: (1) the mediator was significantly associated with sex; (2) sex was significantly associated with dementia; (3) the mediator was significantly associated with dementia; and (4) the association between sex and dementia was attenuated by the mediator. The mediation effects of main groups of factors combined were also tested.

**Table S1. Codes for international classification disease and self-reported fields for dementia**

| **Type of dementia** | **ICD-9*** | **ICD-10** | **Self-reported fields** |
| --- | --- | --- | --- |
| All-cause dementia | 331.0, 290.4, 331.1, 290.2, 290.3, 291.2, 294.1, 331.2, 331.5 | F00, F00.0, F00.1, F00.2, F00.9, G30, G30.0, G30.1, G30.8, G30.9, F01, F01.0, F01.1, F01.2, F01.3, F01.8, F01.9, I67.3, F02.0, G31.0, A81.0, F02, F02.1, F02.2, F02.3, F02.4, F02.8, F03, F05.1, F10.6, G31.1, G31.8 | 1263 |
| Alzheimer’s disease | 331.0 | F00, F00.0, F00.1, F00.2, F00.9, G30, G30.0, G30.1, G30.8, G30.9 |  |
| Vascular dementia | 290.4 | F01, F01.0, F01.1, F01.2, F01.3, F01.8, F01.9 |  |

**Primary and secondary diagnosis from hospital records were used to identify dementia cases.*

**Table S2. Chronic conditions used to create the multimorbidity score for dementia**

| Number | Condition |  |
| --- | --- | --- |
| 1 | Hearing impairment |  |
| 2 | Obesity |  |
| 3 | Hypertension |  |
| 4 | High cholesterol |  |
| 5 | Coronary heart disease |  |
| 6 | Atrial Fibrillation |  |
| 7 | Heart failure |  |
| 8 | Stroke |  |
| 9 | Peripheral vascular disease |  |
| 10 | Other cardiac problem |  |
| 11 | Diabetes |  |
| 12 | COPD |  |
| 13 | Asthma |  |
| 14 | Bronchiectasis |  |
| 15 | Depression |  |
| 16 | Anxiety |  |
| 17 | Schizophrenia |  |
| 18 | Parkinson’s disease |  |
| 19 | Multiple Sclerosis |  |
| 20 | Alcohol problems |  |
| 21 | Psychoactive substance abuse |  |
| 22 | Migraine |  |
| 23 | Epilepsy |  |
| 24 | Painful conditions |  |
| 25 | Chronic fatigue syndrome |  |
| 26 | Connective tissue disorders |  |
| 27 | Osteoporosis |  |
| 28 | Fracture |  |
| 29 | Anorexia |  |
| 30 | Dyspepsia |  |
| 31 | Treated constipation |  |
| 32 | Pernicious anaemia |  |
| 33 | Chronic kidney disease |  |
| 34 | Diverticular disease |  |
| 35 | Inflammatory bowel disease | |
| 36 | Irritable bowel syndrome |  |
| 37 | Viral hepatitis |  |
| 38 | Chronic liver disease |  |
| 39 | Prostate disorders |  |
| 40 | Endometriosis |  |
| 41 | Polycystic ovary |  |
| 42 | Thyroid disorders |  |
| 43 | Eczema |  |
| 44 | HIV |  |
| 45 | Chronic sinusitis |  |
| 46 | Meniere’s disease |  |
| 47 | Glaucoma |  |
| 48 | Cataract |  |
| 49 | AMD |  |
| 50 | Lung cancer |  |
| 51 | Skin cancer |  |
| 52 | Melanoma |  |
| 53 | Stomach cancer |  |
| 54 | Oesophageal cancer |  |
| 55 | Colon cancer |  |
| 56 | Rectal cancer |  |
| 57 | Prostate cancer |  |
| 58 | Ovarian cancer |  |
| 59 | Breast cancer |  |
| 60 | Uterine cancer |  |
| 61 | Other cancers |  |

**Table S3. Potential mediators tested in the analysis**

| Variable | Group |
| --- | --- |
| Education | Socioeconomic factors |
| Household income (pounds) | Socioeconomic factors |
| Townsend index | Socioeconomic factors |
| Alcohol consumption | Lifestyle factors |
| Smoking | Lifestyle factors |
| Physical activity (MET-minutes/week) | Lifestyle factors |
| Diet score | Lifestyle factors |
| Sleep duration (hours) | Lifestyle factors |
| Vitamin B supplement | Lifestyle factors |
| Vitamin C supplement | Lifestyle factors |
| Vitamin D supplement | Lifestyle factors |
| Vitamin E supplement | Lifestyle factors |
| Folate supplement | Lifestyle factors |
| Glucosamine supplement | Lifestyle factors |
| Calcium supplement | Lifestyle factors |
| Zinc supplement | Lifestyle factors |
| Iron supplement | Lifestyle factors |
| Selenium supplement | Lifestyle factors |
| Basal metabolic rate (J/day) | Lifestyle factors |
| BMI (kg/m^2^) | Lifestyle factors |
| Trunk fat percentage | Lifestyle factors |
| Whole body fat percentage | Lifestyle factors |
| Overall health rating | Health related conditions |
| Long-standing illness, disability or infirmity | Health related conditions |
| Multimorbidity risk score | Health related conditions |
| Father's history of heart disease | Familial history of medication conditions |
| Father's history of stroke | Familial history of medication conditions |
| Father's history of cancer | Familial history of medication conditions |
| Father's history of hypertension | Familial history of medication conditions |
| Father's history of diabetes | Familial history of medication conditions |
| Father's history of dementia | Familial history of medication conditions |
| Father's history of Parkinson's disease | Familial history of medication conditions |
| Father's history of depression | Familial history of medication conditions |
| Mother's history of heart disease | Familial history of medication conditions |
| Mother's history of stroke | Familial history of medication conditions |
| Mother's history of cancer | Familial history of medication conditions |
| Mother's history of hypertension | Familial history of medication conditions |
| Mother's history of diabetes | Familial history of medication conditions |
| Mother's history of dementia | Familial history of medication conditions |
| Mother's history of Parkinson's disease | Familial history of medication conditions |
| Mother's history of depression | Familial history of medication conditions |
| Siblings' history of heart disease | Familial history of medication conditions |
| Siblings' history of stroke | Familial history of medication conditions |
| Siblings' history of cancer | Familial history of medication conditions |
| Siblings' history of hypertension | Familial history of medication conditions |
| Siblings' history of diabetes | Familial history of medication conditions |
| Siblings' history of dementia | Familial history of medication conditions |
| Siblings' history of Parkinson's disease | Familial history of medication conditions |
| Siblings' history of depression | Familial history of medication conditions |
| APOE4 | Blood biomarkers |
| HbA1c (mmol/mol) | Blood biomarkers |
| Triglycerides (mmol/L) | Blood biomarkers |
| HDL-C (mmol/L) | Blood biomarkers |
| LDL-C (mmol/L) | Blood biomarkers |
| Haematocrit percentage | Blood biomarkers |
| Mean corpuscular volume (femtolitres) | Blood biomarkers |
| Mean corpuscular haemoglobin concentration (grams/decilitre) | Blood biomarkers |
| Red blood cell count (10^12^ cells/L) | Blood biomarkers |
| Lymphocyte count (10^9^ cells/L) | Blood biomarkers |
| Platelet count (10^9^ cells/L) | Blood biomarkers |
| Platelet crit (%) | Blood biomarkers |
| Mean platelet volume (femtolitres) | Blood biomarkers |
| Platelet distribution width (%) | Blood biomarkers |
| Monocyte count (10^9^ cells/L) | Blood biomarkers |
| Neutrophill count (10^9^ cells/L) | Blood biomarkers |
| Eosinophill count (10^9^ cells/L) | Blood biomarkers |
| Basophill count (10^9^ cells/L) | Blood biomarkers |
| Lymphocyte percentage (%) | Blood biomarkers |
| Monocyte percentage (%) | Blood biomarkers |
| Neutrophill percentage (%) | Blood biomarkers |
| Eosinophill percentage (%) | Blood biomarkers |
| Basophill percentage (%) | Blood biomarkers |
| Nucleated red blood cell percentage (%) | Blood biomarkers |
| Reticulocyte percentage (%) | Blood biomarkers |
| Reticulocyte count (10^12^ cells/L) | Blood biomarkers |
| Mean reticulocyte volume (femtolitres) | Blood biomarkers |
| Mean sphered cell volume (femtolitres) | Blood biomarkers |
| Immature reticulocyte fraction (%) | Blood biomarkers |
| High light scatter reticulocyte percentage (%) | Blood biomarkers |
| High light scatter reticulocyte count (10^12^ cells/L) | Blood biomarkers |
| Albumin (g/L) | Blood biomarkers |
| Alkaline phosphatase (U/L) | Blood biomarkers |
| Alanine aminotransferase (U/L) | Blood biomarkers |
| Apolipoprotein A (g/L) | Blood biomarkers |
| Apolipoprotein B (g/L) | Blood biomarkers |
| Direct bilirubin (umol/L) | Blood biomarkers |
| Urea (mmol/L) | Blood biomarkers |
| Creatinine (umol/L) | Blood biomarkers |
| C-reactive protein (mg/L) | Blood biomarkers |
| Gamma glutamyltransferase (U/L) | Blood biomarkers |
| IGF-1 (nmol/L) | Blood biomarkers |
| Lipoprotein A (nmol/L) | Blood biomarkers |
| Phosphate (mmol/L) | Blood biomarkers |
| SHBG (nmol/L) | Blood biomarkers |
| Total bilirubin (umol/L) | Blood biomarkers |
| Testosterone (nmol/L) | Blood biomarkers |
| Urate (umol/L) | Blood biomarkers |
| Vitamin D (nmol/L) | Blood biomarkers |
| Microalbumin in urine (mg/L) | Urinary biomarkers |
| Creatinine in urine (micromole/L) | Urinary biomarkers |
| Potassium in urine (micromole/L) | Urinary biomarkers |
| Sodium in urine (micromole/L) | Urinary biomarkers |
| Nitrogen dioxide air pollution 2010 (micro-g/m^3^) | Pollution measures |
| Nitrogen oxides air pollution 2010 (micro-g/m^3^) | Pollution measures |
| Particulate matter air pollution 2010 (micro-g/m^3^) | Pollution measures |
| Particulate matter air pollution 2010 (micro-g/m^3^) | Pollution measures |
| Particulate matter air pollution absorbance 2010 (micro-g/m^3^) | Pollution measures |
| Particulate matter air pollution 2.5-10um 2010 (micro-g/m^3^) | Pollution measures |
| Traffic intensity on the nearest road (average total number of motor vehicles per 24 hours) | Pollution measures |
| Inverse distance to the nearest road | Pollution measures |
| Total traffic load on major roads (average total number of motor vehicles per 24 hours) | Pollution measures |
| Traffic intensity on the nearest major road (average total number of motor vehicles per 24 hours) | Pollution measures |
| Total traffic load (average total number of motor vehicles per 24 hours) | Pollution measures |
| Close to major road | Pollution measures |
| Sum of road length of major roads within 100m | Pollution measures |
| Nitrogen dioxide air pollution 2005 (micro-g/m^3^) | Pollution measures |
| Nitrogen dioxide air pollution 2006 (micro-g/m^3^) | Pollution measures |
| Nitrogen dioxide air pollution 2007 (micro-g/m^3^) | Pollution measures |
| Particulate matter air pollution 2007 (micro-g/m^3^) | Pollution measures |
| Average daytime sound level of noise pollution (dB) | Pollution measures |
| Average evening sound level of noise pollution (dB) | Pollution measures |
| Average night-time sound level of noise pollution (dB) | Pollution measures |
| Average 16-hour sound level of noise pollution (dB) | Pollution measures |
| Average 24-hour sound level of noise pollution (dB) | Pollution measures |
| Greenspace percentage, buffer 1000m | Pollution measures |
| Domestic garden percentage, buffer 1000m | Pollution measures |
| Water percentage, buffer 1000m | Pollution measures |
| Greenspace percentage, buffer 300m | Pollution measures |
| Domestic garden percentage, buffer 300m | Pollution measures |
| Water percentage, buffer 300m | Pollution measures |
| Natural environment percentage, buffer 1000m | Pollution measures |
| Natural environment percentage, buffer 300m | Pollution measures |

**Table S4.** **Other baseline characteristics in women and men**

|  | Women | Men | P-value* |
| --- | --- | --- | --- |
| Vitamin B supplement |  |  | 0.83 |
| No | 212331 (98.9) | 212316 (98.9) |  |
| Yes | 2339 (1.1) | 2354 (1.1) |  |
| Vitamin C supplement |  |  | <0.0001 |
| No | 209161 (97.4) | 210222 (97.9) |  |
| Yes | 5509 (2.6) | 4448 (2.1) |  |
| Vitamin D supplement |  |  | <0.0001 |
| No | 209415 (97.6) | 211051 (98.3) |  |
| Yes | 5255 (2.4) | 3619 (1.7) |  |
| Vitamin E supplement |  |  | <0.0001 |
| No | 209279 (97.5) | 210731 (98.2) |  |
| Yes | 5391 (2.5) | 3939 (1.8) |  |
| Folate supplement |  |  | <0.0001 |
| No | 212016 (98.8) | 213096 (99.3) |  |
| Yes | 2654 (1.2) | 1574 (0.7) |  |
| Glucosamine supplement |  |  | <0.0001 |
| No | 184768 (86.1) | 192615 (89.7) |  |
| Yes | 29902 (13.9) | 22055 (10.3) |  |
| Calcium supplement |  |  | <0.0001 |
| No | 199796 (93.1) | 211016 (98.3) |  |
| Yes | 14874 (6.9) | 3654 (1.7) |  |
| Zinc supplement |  |  | <0.0001 |
| No | 206548 (96.2) | 209186 (97.4) |  |
| Yes | 8122 (3.8) | 5484 (2.6) |  |
| Iron supplement |  |  | <0.0001 |
| No | 209360 (97.5) | 211420 (98.5) |  |
| Yes | 5310 (2.5) | 3250 (1.5) |  |
| Selenium supplement |  |  | <0.0001 |
| No | 209559 (97.6) | 210821 (98.2) |  |
| Yes | 5111 (2.4) | 3849 (1.8) |  |
| Father's history of heart disease |  |  | <0.0001 |
| No | 150503 (70.1) | 155755 (72.6) |  |
| Yes | 64167 (29.9) | 58915 (27.4) |  |
| Father's history of stroke |  |  | 0.38 |
| No | 185417 (86.4) | 185613 (86.5) |  |
| Yes | 29253 (13.6) | 29057 (13.5) |  |
| Father's history of cancer |  |  | 1 |
| No | 173946 (81.0) | 173937 (81.0) |  |
| Yes | 40724 (19.0) | 40733 (19.0) |  |
| Father's history of hypertension |  |  | <0.0001 |
| No | 171425 (79.9) | 174479 (81.3) |  |
| Yes | 43245 (20.1) | 40191 (18.7) |  |
| Father's history of diabetes |  |  | <0.0001 |
| No | 196389 (91.5) | 197515 (92.0) |  |
| Yes | 18281 (8.5) | 17155 (8.0) |  |
| Father's history of dementia |  |  | <0.0001 |
| No | 205414 (95.7) | 206154 (96.0) |  |
| Yes | 9256 (4.3) | 8516 (4.0) |  |
| Father's history of Parkinson's disease |  |  | <0.0001 |
| No | 209665 (97.7) | 210363 (98.0) |  |
| Yes | 5005 (2.3) | 4307 (2.0) |  |
| Father's history of depression |  |  | <0.0001 |
| No | 206751 (96.3) | 208573 (97.2) |  |
| Yes | 7919 (3.7) | 6097 (2.8) |  |
| Mother's history of heart disease |  |  | <0.0001 |
| No | 170184 (79.3) | 180308 (84.0) |  |
| Yes | 44486 (20.7) | 34362 (16.0) |  |
| Mother's history of stroke |  |  | <0.0001 |
| No | 183692 (85.6) | 189237 (88.2) |  |
| Yes | 30978 (14.4) | 25433 (11.8) |  |
| Mother's history of cancer |  |  | 0.0071 |
| No | 181428 (84.5) | 182064 (84.8) |  |
| Yes | 33242 (15.5) | 32606 (15.2) |  |
| Mother's history of hypertension |  |  | <0.0001 |
| No | 147128 (68.5) | 163058 (76.0) |  |
| Yes | 67542 (31.5) | 51612 (24.0) |  |
| Mother's history of diabetes |  |  | <0.0001 |
| No | 195556 (91.1) | 197073 (91.8) |  |
| Yes | 19114 (8.9) | 17597 (8.2) |  |
| Mother's history of dementia |  |  | <0.0001 |
| No | 196609 (91.6) | 198731 (92.6) |  |
| Yes | 18061 (8.4) | 15939 (7.4) |  |
| Mother's history of Parkinson's disease |  |  | <0.0001 |
| No | 211175 (98.4) | 211650 (98.6) |  |
| Yes | 3495 (1.6) | 3020 (1.4) |  |
| Mother's history of depression |  |  | <0.0001 |
| No | 199667 (93.0) | 204012 (95.0) |  |
| Yes | 15003 (7.0) | 10658 (5.0) |  |
| Siblings' history of heart disease |  |  | <0.0001 |
| No | 195392 (91.0) | 198478 (92.5) |  |
| Yes | 19278 (9.0) | 16192 (7.5) |  |
| Siblings' history of stroke |  |  | <0.0001 |
| No | 208605 (97.2) | 209411 (97.6) |  |
| Yes | 6065 (2.8) | 5259 (2.4) |  |
| Siblings' history of cancer |  |  | <0.0001 |
| No | 196808 (91.7) | 198271 (92.4) |  |
| Yes | 17862 (8.3) | 16399 (7.6) |  |
| Siblings' history of hypertension |  |  | <0.0001 |
| No | 174504 (81.3) | 183918 (85.7) |  |
| Yes | 40166 (18.7) | 30752 (14.3) |  |
| Siblings' history of diabetes |  |  | <0.0001 |
| No | 199205 (92.8) | 201383 (93.8) |  |
| Yes | 15465 (7.2) | 13287 (6.2) |  |
| Siblings' history of dementia |  |  | <0.0001 |
| No | 213550 (99.5) | 213790 (99.6) |  |
| Yes | 1120 (0.5) | 880 (0.4) |  |
| Siblings' history of Parkinson's disease |  |  | 0.0038 |
| No | 213659 (99.5) | 213785 (99.6) |  |
| Yes | 1011 (0.5) | 885 (0.4) |  |
| Siblings' history of depression |  |  | <0.0001 |
| No | 200207 (93.3) | 205366 (95.7) |  |
| Yes | 14463 (6.7) | 9304 (4.3) |  |
| Basal metabolic rate (J/day) | 5644.48 ± 664.89 | 7798.12 ± 1033.37 | <0.0001 |
| Haematocrit percentage | 39.28 ± 2.75 | 43.30 ± 2.96 | <0.0001 |
| Mean corpuscular volume (femtolitres) | 91.07 ± 4.38 | 91.61 ± 4.23 | <0.0001 |
| Mean corpuscular haemoglobin concentration (grams/decilitre) | 31.36 ± 1.83 | 31.75 ± 1.74 | <0.0001 |
| Red blood cell count (10^12^ cells/L) | 4.32 ± 0.33 | 4.73 ± 0.37 | <0.0001 |
| Lymphocyte count (10^9^ cells/L) | 2.01 ± 1.02 | 1.90 ± 1.33 | <0.0001 |
| Platelet count (10^9^ cells/L) | 265.75 ± 58.75 | 238.27 ± 55.03 | <0.0001 |
| Platelet crit (%) | 0.25 ± 0.05 | 0.22 ± 0.04 | <0.0001 |
| Mean platelet volume (femtolitres) | 9.36 ± 1.07 | 9.28 ± 1.05 | <0.0001 |
| Platelet distribution width (%) | 16.43 ± 0.49 | 16.57 ± 0.52 | <0.0001 |
| Monocyte count (10^9^ cells/L) | 0.44 ± 0.32 | 0.52 ± 0.22 | <0.0001 |
| Neutrophill count (10^9^ cells/L) | 4.22 ± 1.37 | 4.29 ± 1.41 | <0.0001 |
| Eosinophill count (10^9^ cells/L) | 0.16 ± 0.13 | 0.19 ± 0.14 | <0.0001 |
| Basophill count (10^9^ cells/L) | 0.04 ± 0.05 | 0.03 ± 0.05 | <0.0001 |
| Lymphocyte percentage (%) | 29.57 ± 7.10 | 27.61 ± 7.22 | <0.0001 |
| Monocyte percentage (%) | 6.60 ± 2.48 | 7.67 ± 2.72 | <0.0001 |
| Neutrophill percentage (%) | 60.84 ± 8.11 | 61.45 ± 8.38 | <0.0001 |
| Eosinophill percentage (%) | 2.40 ± 1.74 | 2.74 ± 1.87 | <0.0001 |
| Basophill percentage (%) | 0.59 ± 0.65 | 0.54 ± 0.52 | <0.0001 |
| Nucleated red blood cell percentage (%) | 0.033 ± 0.413 | 0.026 ± 0.461 | <0.0001 |
| Reticulocyte percentage (%) | 1.32 ± 0.90 | 1.38 ± 0.88 | <0.0001 |
| Reticulocyte count (10^12^ cells/L) | 0.06 ± 0.04 | 0.07 ± 0.04 | <0.0001 |
| Mean reticulocyte volume (femtolitres) | 105.50 ± 7.58 | 106.31 ± 7.60 | <0.0001 |
| Mean sphered cell volume (femtolitres) | 83.00 ± 5.13 | 82.78 ± 5.17 | <0.0001 |
| Immature reticulocyte fraction (%) | 0.29 ± 0.06 | 0.29 ± 0.06 | 0.57 |
| High light scatter reticulocyte percentage (%) | 0.39 ± 0.38 | 0.41 ± 0.29 | <0.0001 |
| High light scatter reticulocyte count (10^12^ cells/L) | 0.017 ± 0.009 | 0.019 ± 0.010 | <0.0001 |
| Microalbumin in urine (mg/L) | 23.47 ± 66.70 | 34.31 ± 97.42 | <0.0001 |
| Creatinine in urine (micromole/L) | 7116.97 ± 4822.89 | 10825.36 ± 5994.04 | <0.0001 |
| Potassium in urine (micromole/L) | 59.25 ± 32.88 | 68.60 ± 33.65 | <0.0001 |
| Sodium in urine (micromole/L) | 66.58 ± 39.32 | 87.91 ± 44.87 | <0.0001 |
| Albumin (g/L) | 44.94 ± 2.45 | 45.54 ± 2.49 | <0.0001 |
| Alkaline phosphatase (U/L) | 85.06 ± 26.63 | 82.03 ± 24.52 | <0.0001 |
| Alanine aminotransferase (U/L) | 20.30 ± 11.86 | 27.41 ± 14.85 | <0.0001 |
| Apolipoprotein A (g/L) | 1.63 ± 0.26 | 1.45 ± 0.22 | <0.0001 |
| Apolipoprotein B (g/L) | 1.04 ± 0.23 | 1.03 ± 0.23 | <0.0001 |
| Direct bilirubin (umol/L) | 1.49 ± 0.68 | 1.96 ± 0.91 | <0.0001 |
| Urea (mmol/L) | 5.28 ± 1.30 | 5.63 ± 1.41 | <0.0001 |
| Creatinine (umol/L) | 64.42 ± 12.98 | 81.56 ± 17.56 | <0.0001 |
| C-reactive protein (mg/L) | 2.72 ± 4.29 | 2.48 ± 4.30 | <0.0001 |
| Gamma glutamyltransferase (U/L) | 30.25 ± 33.18 | 45.84 ± 47.82 | <0.0001 |
| IGF-1 (nmol/L) | 20.87 ± 5.59 | 21.92 ± 5.41 | <0.0001 |
| Lipoprotein A (nmol/L) | 44.58 ± 44.12 | 43.41 ± 43.97 | <0.0001 |
| Phosphate (mmol/L) | 1.19 ± 0.14 | 1.12 ± 0.15 | <0.0001 |
| SHBG (nmol/L) | 62.16 ± 28.91 | 40.20 ± 16.57 | <0.0001 |
| Total bilirubin (umol/L) | 8.14 ± 3.60 | 10.32 ± 4.76 | <0.0001 |
| Testosterone (nmol/L) | 1.08 ± 0.91 | 11.99 ± 3.60 | <0.0001 |
| Urate (umol/L) | 271.34 ± 64.75 | 354.60 ± 70.08 | <0.0001 |
| Vitamin D (nmol/L) | 49.24 ± 19.97 | 49.32 ± 20.38 | 0.17 |
| Nitrogen dioxide air pollution 2010 (micro-g/m^3^) | 26.34 ± 7.34 | 26.43 ± 7.47 | <0.0001 |
| Nitrogen oxides air pollution 2010 (micro-g/m^3^) | 43.48 ± 14.93 | 43.72 ± 15.32 | <0.0001 |
| Particulate matter air pollution 2010 (micro-g/m^3^) | 16.19 ± 1.85 | 16.20 ± 1.87 | 0.0476 |
| Particulate matter air pollution 2010 (micro-g/m^3^) | 9.98 ± 1.03 | 10.00 ± 1.05 | <0.0001 |
| Particulate matter air pollution absorbance 2010 (micro-g/m^3^) | 1.17 ± 0.26 | 1.17 ± 0.26 | <0.0001 |
| Particulate matter air pollution 2.5-10um 2010 (micro-g/m^3^) | 6.40 ± 0.88 | 6.41 ± 0.89 | 0.0005 |
| Traffic intensity on the nearest road (average total number of motor vehicles per 24 hours) | 1431.48 ± 4406.22 | 1462.57 ± 4464.23 | 0.0217 |
| Inverse distance to the nearest road | 0.05 ± 0.07 | 0.05 ± 0.07 | 0.69 |
| Total traffic load on major roads (average total number of motor vehicles per 24 hours) | 23536.86 ± 21155.31 | 23557.13 ± 21139.14 | 0.75 |
| Traffic intensity on the nearest major road (average total number of motor vehicles per 24 hours) | 0.01 ± 0.10 | 0.01 ± 0.11 | 0.16 |
| Total traffic load (average total number of motor vehicles per 24 hours) | 361400.8 ± 1081406 | 372990.9 ± 1093117 | 0.0005 |
| Close to major road | 0.068 ± 0.250 | 0.071 ± 0.255 | <0.0001 |
| Sum of road length of major roads within 100m | 26.83 ± 74.35 | 28.07 ± 76.87 | <0.0001 |
| Nitrogen dioxide air pollution 2005 (micro-g/m^3^) | 29.31 ± 9.59 | 29.40 ± 9.67 | 0.0012 |
| Nitrogen dioxide air pollution 2006 (micro-g/m^3^) | 28.42 ± 8.75 | 28.51 ± 8.81 | 0.0011 |
| Nitrogen dioxide air pollution 2007 (micro-g/m^3^) | 30.07 ± 10.14 | 30.14 ± 10.19 | 0.0230 |
| Particulate matter air pollution 2007 (micro-g/m^3^) | 21.94 ± 2.77 | 21.94 ± 2.76 | 0.56 |
| Average daytime sound level of noise pollution (dB) | 55.35 ± 4.17 | 55.41 ± 4.24 | <0.0001 |
| Average evening sound level of noise pollution (dB) | 51.60 ± 4.17 | 51.66 ± 4.24 | <0.0001 |
| Average night-time sound level of noise pollution (dB) | 46.53 ± 4.17 | 46.59 ± 4.24 | <0.0001 |
| Average 16-hour sound level of noise pollution (dB) | 54.41 ± 4.17 | 54.47 ± 4.24 | <0.0001 |
| Average 24-hour sound level of noise pollution (dB) | 55.99 ± 4.17 | 56.05 ± 4.24 | <0.0001 |
| Greenspace percentage, buffer 1000m | 45.66 ± 21.39 | 45.68 ± 21.33 | 0.80 |
| Domestic garden percentage, buffer 1000m | 24.60 ± 11.15 | 24.32 ± 11.12 | <0.0001 |
| Water percentage, buffer 1000m | 1.21 ± 2.39 | 1.22 ± 2.39 | 0.0266 |
| Greenspace percentage, buffer 300m | 35.54 ± 23.04 | 35.61 ± 22.96 | 0.37 |
| Domestic garden percentage, buffer 300m | 31.67 ± 14.44 | 31.38 ± 14.48 | <0.0001 |
| Water percentage, buffer 300m | 0.84 ± 2.81 | 0.86 ± 2.84 | 0.0132 |
| Natural environment percentage, buffer 1000m | 41.66 ± 25.51 | 41.61 ± 25.50 | 0.46 |
| Natural environment percentage, buffer 300m | 26.85 ± 25.26 | 26.88 ± 25.24 | 0.72 |

Data are mean (standard deviation), or N (%). IGF-1: insulin-like growth factor 1; SHBG, sex hormone-binding globulin.

^*^T-test was used to test the difference of continuous variables between genders and Chi-square for categorical variables.

**Table S5. Categorical variables in imputed and non-imputed data**

|  | Imputed data | Non-imputed data |
| --- | --- | --- |
| APOE4 carrier* |  |  |
| No | 327738(76.3) | 317626(74.0) |
| Yes | 101602(23.7) | 100492(23.4) |
| Missing |  | 11222(2.6) |
| Education |  |  |
| College/university degree | 138419(32.2) | 135818(31.6) |
| Upper secondary | 48676(11.3) | 47398(11.0) |
| Final stage of secondary education | 92542(21.6) | 90649(21.1) |
| Lower secondary | 22879(5.3) | 22676(5.3) |
| First stage of secondary education | 28816(6.7) | 28686(6.7) |
| Vocational qualifications | 21917(5.1) | 21898(5.1) |
| None of above | 76091(17.7) | 75118(17.5) |
| Missing |  | 7097(1.7) |
| Household income (pounds) |  |  |
| <18,000 | 96719(22.5) | 82626(19.2) |
| 18,000-30,999 | 118507(27.6) | 93837(21.9) |
| 31,000-51,999 | 113119(26.3) | 95968(22.4) |
| 52,000-100,000 | 80898(18.8) | 74968(17.5) |
| >100,000 | 20097(4.7) | 19925(4.6) |
| Missing |  | 62016(14.5) |
| Alcohol consumption |  |  |
| Never | 13653(3.2) | 13651(3.2) |
| Previous | 14967(3.5) | 14957(3.5) |
| Current | 400720(93.3) | 400352(93.2) |
| Missing |  | 380(0.1) |
| Smoking |  |  |
| Never | 229500(53.5) | 228770(53.3) |
| Former | 154550(36.0) | 153809(35.8) |
| Current | 45290(10.5) | 45237(10.5) |
| Missing |  | 1524(0.4) |
| Overall health rating |  |  |
| Excellent | 71165(16.6) | 71131(16.6) |
| Good | 249750(58.2) | 248802(57.9) |
| Fair | 89641(20.9) | 89019(20.7) |
| Poor | 18784(4.4) | 18707(4.4) |
| Missing |  | 1681(0.4) |
| Long-standing illness, disability or infirmity |  |  |
| No | 287197(66.9) | 281228(65.5) |
| Yes | 142143(33.1) | 138220(32.2) |
| Missing |  | 9892(2.3) |

Data are N (%). APOE4, apolipoprotein E4.

^*^APOE4+ dominant model of E3/E4 and E4/E4 was used to define the presence of APOE4.

**Table S6. Continuous variables in imputed and non-imputed data**

|  | Imputed data | |  | Non-imputed data | |
| --- | --- | --- | --- | --- | --- |
|  | N | Mean±SD |  | N | Mean±SD |
| Townsend index | 429340 | -1.45±3.00 |  | 428839 | -1.45±3.00 |
| Physical activity (MET-minutes/week) | 429340 | 2560.53±2178.50 |  | 380377 | 2665.87±2717.54 |
| Diet score | 429340 | 3.85±1.44 |  | 428576 | 3.85±1.44 |
| Sleep duration (hours) | 429340 | 7.17±1.09 |  | 429340 | 7.12±1.25 |
| BMI (kg/m^2^) | 429340 | 27.44±4.67 |  | 421214 | 27.45±4.72 |
| Multimorbidity risk score | 429340 | 0.27±0.3 |  | 429340 | 0.27±0.3 |
| Trunk fat percentage | 420962 | 30.88±7.96 |  | 420962 | 30.88±7.96 |
| Whole body fat percentage | 420960 | 30.94±8.51 |  | 420960 | 30.94±8.51 |
| HbA1c (mmol/mol) | 429340 | 14.26±1.22 |  | 409657 | 14.26±1.24 |
| Triglycerides (mmol/L) | 429340 | 1.77±1 |  | 401779 | 1.77±1.04 |
| HDL-C (mmol/L) | 429340 | 1.44±0.35 |  | 368427 | 1.44±0.38 |
| LDL-C (mmol/L) | 429340 | 3.56±0.84 |  | 401339 | 3.56±0.87 |
| Basal metabolic rate (J/day) | 421205 | 6719.7±1383.49 |  | 421205 | 6719.7±1383.49 |
| Haematocrit percentage | 429340 | 41.29±3.49 |  | 409657 | 41.3±3.54 |
| Mean corpuscular volume (femtolitres) | 429340 | 91.34±4.32 |  | 409655 | 91.34±4.4 |
| Mean corpuscular haemoglobin concentration (grams/decilitre) | 429340 | 31.56±1.8 |  | 409654 | 31.56±1.83 |
| Red blood cell count (10^12^ cells/L) | 429340 | 4.53±0.41 |  | 409657 | 4.53±0.41 |
| Lymphocyte count (10^9^ cells/L) | 429340 | 1.95±1.18 |  | 408910 | 1.95±1.21 |
| Platelet count (10^9^ cells/L) | 429340 | 252.01±58.55 |  | 409655 | 251.92±59.59 |
| Platelet crit (%) | 429340 | 0.23±0.05 |  | 409652 | 0.23±0.05 |
| Mean platelet volume (femtolitres) | 429340 | 9.32±1.06 |  | 409652 | 9.32±1.08 |
| Platelet distribution width (%) | 429340 | 16.5±0.51 |  | 409652 | 16.5±0.52 |
| Monocyte count (10^9^ cells/L) | 429340 | 0.48±0.28 |  | 408910 | 0.48±0.28 |
| Neutrophill count (10^9^ cells/L) | 429340 | 4.25±1.39 |  | 408910 | 4.25±1.42 |
| Eosinophill count (10^9^ cells/L) | 429340 | 0.17±0.13 |  | 408910 | 0.17±0.14 |
| Basophill count (10^9^ cells/L) | 429340 | 0.03±0.05 |  | 408910 | 0.03±0.05 |
| Lymphocyte percentage (%) | 429340 | 28.59±7.23 |  | 408916 | 28.58±7.37 |
| Monocyte percentage (%) | 429340 | 7.13±2.66 |  | 408916 | 7.14±2.71 |
| Neutrophill percentage (%) | 429340 | 61.15±8.25 |  | 408916 | 61.15±8.41 |
| Eosinophill percentage (%) | 429340 | 2.57±1.82 |  | 408916 | 2.57±1.85 |
| Basophill percentage (%) | 429340 | 0.57±0.59 |  | 408916 | 0.57±0.6 |
| Nucleated red blood cell percentage (%) | 429340 | 0.029±0.437 |  | 408897 | 0.029±0.394 |
| Reticulocyte percentage (%) | 429340 | 1.35±0.89 |  | 402777 | 1.35±0.9 |
| Reticulocyte count (10^12^ cells/L) | 429340 | 0.06±0.04 |  | 402777 | 0.06±0.04 |
| Mean reticulocyte volume (femtolitres) | 429340 | 105.9±7.6 |  | 402777 | 105.89±7.78 |
| Mean sphered cell volume (femtolitres) | 429340 | 82.89±5.15 |  | 402778 | 82.87±5.26 |
| Immature reticulocyte fraction (%) | 429340 | 0.29±0.06 |  | 402777 | 0.29±0.06 |
| High light scatter reticulocyte percentage (%) | 429340 | 0.4±0.34 |  | 402778 | 0.4±0.35 |
| High light scatter reticulocyte count (10^12^ cells/L) | 429340 | 0.02±0.01 |  | 402777 | 0.02±0.01 |
| Microalbumin in urine (mg/L) | 429340 | 28.89±83.66 |  | 130921 | 30.41±123.87 |
| Creatinine in urine (micromole/L) | 429340 | 8971.17±5747.38 |  | 414631 | 8968.26±5809.46 |
| Potassium in urine (micromole/L) | 429340 | 63.93±33.59 |  | 413760 | 63.84±33.96 |
| Sodium in urine (micromole/L) | 429340 | 77.24±43.51 |  | 413788 | 77.3±44.01 |
| Albumin (g/L) | 429340 | 45.24±2.49 |  | 368601 | 45.24±2.62 |
| Alkaline phosphatase (U/L) | 429340 | 83.54±25.64 |  | 402116 | 83.52±26.27 |
| Alanine aminotransferase (U/L) | 429340 | 23.86±13.9 |  | 401941 | 23.85±14.21 |
| Apolipoprotein A (g/L) | 429340 | 1.54±0.26 |  | 366460 | 1.53±0.27 |
| Apolipoprotein B (g/L) | 429340 | 1.03±0.23 |  | 400079 | 1.03±0.24 |
| Direct bilirubin (umol/L) | 429340 | 1.73±0.83 |  | 344625 | 1.85±0.85 |
| Urea (mmol/L) | 429340 | 5.45±1.37 |  | 401813 | 5.45±1.4 |
| Creatinine (umol/L) | 429340 | 72.99±17.66 |  | 401896 | 73.03±18 |
| C-reactive protein (mg/L) | 429340 | 2.6±4.3 |  | 401244 | 2.59±4.4 |
| Gamma glutamyltransferase (U/L) | 429340 | 38.05±41.89 |  | 401878 | 38.02±42.76 |
| IGF-1 (nmol/L) | 429340 | 21.39±5.53 |  | 399904 | 21.4±5.67 |
| Lipoprotein A (nmol/L) | 429340 | 44±44.05 |  | 320197 | 43.99±49.29 |
| Phosphate (mmol/L) | 429340 | 1.16±0.15 |  | 367886 | 1.16±0.16 |
| SHBG (nmol/L) | 429340 | 51.18±25.99 |  | 365009 | 50.98±27.23 |
| Total bilirubin (umol/L) | 429340 | 9.23±4.36 |  | 400368 | 9.23±4.46 |
| Testosterone (nmol/L) | 429340 | 6.54±6.06 |  | 366548 | 7.04±6.09 |
| Urate (umol/L) | 429340 | 312.97±79.28 |  | 401622 | 313.07±80.64 |
| Vitamin D (nmol/L) | 429340 | 49.28±20.17 |  | 384904 | 49.56±20.95 |
| Nitrogen dioxide air pollution 2010 (micro-g/m^3^) | 429340 | 26.39±7.4 |  | 423245 | 26.36±7.41 |
| Nitrogen oxides air pollution 2010 (micro-g/m^3^) | 429340 | 43.6±15.13 |  | 423245 | 43.55±15.15 |
| Particulate matter air pollution 2010 (micro-g/m^3^) | 429340 | 16.19±1.86 |  | 393223 | 16.19±1.89 |
| Particulate matter air pollution 2010 (micro-g/m^3^) | 429340 | 9.99±1.04 |  | 393223 | 9.96±1.05 |
| Particulate matter air pollution absorbance 2010 (micro-g/m^3^) | 429340 | 1.17±0.26 |  | 393223 | 1.17±0.26 |
| Particulate matter air pollution 2.5-10um 2010 (micro-g/m^3^) | 429340 | 6.4±0.88 |  | 393223 | 6.41±0.9 |
| Traffic intensity on the nearest road (average total number of motor vehicles per 24 hours) | 429340 | 1447.02±4435.34 |  | 423245 | 1446.1±4460.54 |
| Inverse distance to the nearest road | 429340 | 0.05±0.07 |  | 423245 | 0.05±0.07 |
| Total traffic load on major roads (average total number of motor vehicles per 24 hours) | 429340 | 23547±21147.21 |  | 423245 | 23548.83±21267.26 |
| Traffic intensity on the nearest major road (average total number of motor vehicles per 24 hours) | 429340 | 0.01±0.1 |  | 423245 | 0.01±0.1 |
| Total traffic load (average total number of motor vehicles per 24 hours) | 429340 | 367195.83±1087291.15 |  | 423245 | 366386.12±1093410.83 |
| Close to major road | 429340 | 0.07±0.25 |  | 423245 | 0.07±0.25 |
| Sum of road length of major roads within 100m | 429340 | 27.45±75.62 |  | 423245 | 27.38±76.05 |
| Nitrogen dioxide air pollution 2005 (micro-g/m^3^) | 429340 | 29.35±9.63 |  | 423245 | 29.31±9.65 |
| Nitrogen dioxide air pollution 2006 (micro-g/m^3^) | 429340 | 28.46±8.78 |  | 423245 | 28.43±8.8 |
| Nitrogen dioxide air pollution 2007 (micro-g/m^3^) | 429340 | 30.1±10.16 |  | 423245 | 30.06±10.19 |
| Particulate matter air pollution 2010 (micro-g/m^3^) | 429340 | 21.94±2.77 |  | 422249 | 21.93±2.78 |
| Average daytime sound level of noise pollution (dB) | 429340 | 55.38±4.21 |  | 423245 | 55.37±4.23 |
| Average evening sound level of noise pollution (dB) | 429340 | 51.63±4.21 |  | 423245 | 51.63±4.23 |
| Average night-time sound level of noise pollution (dB) | 429340 | 46.56±4.21 |  | 423245 | 46.55±4.23 |
| Average 16-hour sound level of noise pollution (dB) | 429340 | 54.44±4.21 |  | 423245 | 54.44±4.23 |
| Average 24-hour sound level of noise pollution (dB) | 429340 | 56.02±4.21 |  | 423245 | 56.02±4.23 |
| Greenspace percentage, buffer 1000m | 429340 | 45.67±21.36 |  | 375301 | 45.96±21.6 |
| Domestic garden percentage, buffer 1000m | 429340 | 24.46±11.14 |  | 375301 | 24.11±11.29 |
| Water percentage, buffer 1000m | 429340 | 1.21±2.39 |  | 375301 | 1.28±2.5 |
| Greenspace percentage, buffer 300m | 429340 | 35.57±23 |  | 375301 | 36.03±23.39 |
| Domestic garden percentage, buffer 300m | 429340 | 31.52±14.46 |  | 375301 | 31.1±14.77 |
| Water percentage, buffer 300m | 429340 | 0.85±2.83 |  | 375301 | 0.91±2.97 |
| Natural environment percentage, buffer 1000m | 429340 | 41.63±25.51 |  | 425235 | 41.62±25.52 |
| Natural environment percentage, buffer 300m | 429340 | 26.86±25.25 |  | 425235 | 26.84±25.26 |

Data are mean (standard deviation), or N (%). BMI, body mass index; HbA1c, glycated haemoglobin; HDL-C, high-density lipoprotein cholesterol; IGF-1: insulin-like growth factor 1; LDL-C, low-density lipoprotein cholesterol; MET, metabolic equivalent; SHBG, sex hormone-binding globulin.

**Table S7. Risk for incident dementia associated with mediators**

| Mediator | Hazard ratio (95% CI)* |
| --- | --- |
| Townsend index | 1.20 (1.17-1.23) |
| Diet score | 0.93 (0.90-0.95) |
| Glucosamine supplement^†^ |  |
| No | Reference |
| Yes | 0.84 (0.77-0.90) |
| Smoking^†^ |  |
| Never | Reference |
| Former | 1.11 (1.05-1.17) |
| Current | 1.40 (1.28-1.53) |
| BMI | 1.03 (1.01-1.06) |
| Multimorbidity risk score | 1.45 (1.42-1.47) |
| Mother's history of hypertension^†^ |  |
| No | Reference |
| Yes | 0.83 (0.78-0.88) |
| Overall health rating^†^ |  |
| Excellent | Reference |
| Good | 1.39 (1.27-1.53) |
| Fair | 2.28 (2.07-2.51) |
| Poor | 4.56 (4.07-5.10) |
| Long-term illness^†^ |  |
| No | Reference |
| Yes | 1.96 (1.86-2.06) |
| HDL-C | 0.96 (0.93-0.98) |
| LDL-C | 0.89 (0.87-0.92) |
| Mean corpuscular volume | 1.08 (1.05-1.11) |
| Mean corpuscular haemoglobin | 1.03 (1.01-1.06) |
| Neutrophil percentage | 1.13 (1.10-1.16) |
| Mean platelet volume | 0.95 (0.93-0.98) |
| Monocyte count | 1.01 (1.00-1.02) |
| Neutrophil count | 1.12 (1.10-1.14) |
| Lymphocyte percentage | 0.90 (0.87-0.92) |
| Mean reticulocyte volume | 1.08 (1.05-1.11) |
| Creatinine in urine | 1.09 (1.06-1.12) |
| Apolipoprotein A | 0.93 (0.90-0.96) |
| Apolipoprotein B | 0.93 (0.91-0.95) |
| Direct bilirubin | 1.03 (1.01-1.05) |
| Creatinine in blood | 1.02 (1.00-1.05) |
| Gamma glutamyltransferase | 1.07 (1.05-1.09) |
| Alanine aminotransferase | 0.96 (0.93-0.99) |
| Triglycerides | 0.96 (0.93-0.99) |
| Vitamin D | 0.86 (0.83-0.88) |
| Nitrogen oxides air pollution | 1.09 (1.07-1.12) |
| Particulate matter air pollution 2.5-10um | 1.11 (1.08-1.14) |
| Particulate matter air pollution absorbance | 1.08 (1.05-1.10) |

*Cox regression models were used to examine the association between mediators and incident dementia adjusted for age and sex.

^†^Indicate categorical variables where the reference group is notified. For all other variables (continuous), the hazard ratio associated with each standard deviation increment in the mediator was estimated.
